# A novel two-score system for interferon status segregates autoimmune diseases and correlates with clinical features

**DOI:** 10.1038/s41598-018-24198-1

**Published:** 2018-04-11

**Authors:** Y. M. El-Sherbiny, A. Psarras, M. Y. Md Yusof, E. M. A. Hensor, R. Tooze, G. Doody, A. A. A Mohamed, D. McGonagle, M. Wittmann, P. Emery, E. M. Vital

**Affiliations:** 1 National Institute of Health Research Leeds Biomedical Research Centre, Leeds Teaching Hospitals NHS Trust, Leeds, UK; 2 Experimental Haematology, Leeds Institute of Cancer and Pathology, University of Leeds, Leeds, UK; 3 Department of Rheumatology and Rehabilitation, Faculty of Medicine, Assiut University, Asyut, Egypt; 4 Clinical Pathology Department, Faculty of Medicine, Mansoura University, Mansoura, Egypt; 5 Leeds Institute of Rheumatic and Musculoskeletal Medicine, University of Leeds, Leeds, UK

## Abstract

Measurement of type I interferon (IFN-I) has potential to diagnose and stratify autoimmune diseases, but existing results have been inconsistent. Interferon-stimulated-gene (ISG) based methods may be affected by the modularity of the ISG transcriptome, cell-specific expression, response to IFN-subtypes and bimodality of expression. We developed and clinically validated a 2-score system (IFN-Score-A and -B) using Factor Analysis of 31 ISGs measured by TaqMan selected from 3-IFN-annotated modules. We evaluated these scores using *in-vitro* IFN stimulation as well as in sorted cells then clinically validated in a cohort of 328 autoimmune disease patients and healthy controls. ISGs varied in response to IFN-subtypes and both scores varied between cell subsets. IFN-Score-A differentiated Systemic Lupus Erythematosus (SLE) from both Rheumatoid Arthritis (RA) and Healthy Controls (HC) (both p < 0.001), while IFN-Score-B differentiated SLE and RA from HC (both p < 0.001). In SLE, both scores were associated with cutaneous and hematological (all p < 0.05) but not musculoskeletal disease activity. Comparing with bimodal (IFN-high/low) classification, significant differences in IFN-scores were found between diagnostic groups within the IFN-high group. Our continuous 2-score system is more clinically relevant than a simple bimodal classification of IFN status. This system should allow improvement in diagnosis, stratification, and therapy in IFN-mediated autoimmunity.

## Introduction

The type I interferon (IFN-I) family has a central role in antiviral immunity as well as the pathogenesis of several autoimmune diseases: most notably in systemic lupus erythematosus (SLE), but also implicated in rheumatoid arthritis (RA). Many of the strongest genetic susceptibility variants for SLE encode proteins in the innate immune response including molecules involved in endogenous immune stimuli intensification, production of IFN-I, and regulation of cellular response to IFN-I^1,2^. Blockade of IFN-α or the shared IFNAR receptor have both demonstrated efficacy. In RA, IFN-I is found in the synovium and is a biomarker in pre-clinical disease^3,4^. IFN activity is variable within and between autoimmune diseases and may have value in diagnosis, stratifying subtypes of disease and selecting patients for interferon-targeted therapy. However, measurement of IFN-I status is complex and existing IFN-I biomarkers show poor or inconsistent correlations with disease activity^5,6^ as well as response to IFN-I-blocking therapy^7–10^.

Because direct measurement of serum IFN-I proteins is insensitive, IFN-I activity is usually measured indirectly using expression of interferon-stimulated genes (ISGs) as either a “signature”, which usually refers to a categorical variable representing high or low expression of ISGs^11^, or a “score”, which usually refers to a continuous variable derived from level of expression of a set of selected ISGs^12–14^.

ISG-based assays have limitations. First, the group of genes identified as ISGs may not represent a single immune phenomenon. The interferon transcriptome is modular with sets of ISGs described that respond not only to IFN-I but also to IFN-II and -III^15^ as well as non-IFN pathways. Second, gene expression may vary between circulating cell populations^16,17^, and so assays that use whole blood^18–22^ or unsorted PBMCs^23–26^ may show apparent differences in level of ISG expression that are in fact due to changes in the size of cell populations; such changes are characteristic of autoimmune diseases^27^. Lastly, previous IFN scores were bimodal (high or low) but the relative merits of bimodal categorization or continuous analysis have not been compared.

Our objective was to develop and clinically validate continuous Interferon Stimulated Gene Expression (ISG) scores for evaluation of IFN-mediated diseases, accounting for modularity of the ISG transcriptome, cell-specific expression, response to IFN-subtypes and bimodality of expression.

## Results

### Interferon Gene Expression Scoring

We first performed Taqman analysis of 10 genes from each of three IFN-annotated modules previously described, followed by Factor Analysis (FA) to determine whether the gene expression values of multiple genes were driven by a smaller number of unobserved (latent) continuous variables. We included 49 HC (mean (SD) age at sample 31.7 (8.8); 65% female), 114 SLE (45.7 (14.5); 93%), 133 UCTD (47.7 (15.1); 86%) and 32 RA patients (52.2 (15.5); 72%) (total n = 328).

When the FA was performed in the 31 genes, KMO values confirmed the sample adequacy; overall KMO = 0.93 (‘superb’^28^) and KMO values for each of the individual genes all exceeded the acceptable 0.5 limit^28^. Bartlett’s test of sphericity was highly significant [Χ^2^_(465)_ = 11445.2, p < 0.001], indicating sufficiently large correlations between the genes to permit FA. However, The determinant of the correlation matrix was low (<0.00001) when all genes were included, indicating multicollinearity between expression values in the 31 genes. Repeating the factor analysis in a reduced set of 19 genes selected for low squared multiple correlation with the others increased the determinant to 0.00002 but gave very similar results (data not shown) therefore we proceeded with the full gene set.

The parallel analysis indicated that up to 7 factors were present in the data (Fig. S1); however, a simpler solution with fewer cross-loaded items was obtained with 2 factors, which still explained 84% of the variance. Eigenvalues for each of the initial 31 factors and factor loadings (pattern and structure matrix) for each gene are presented in online supplementary Tables S3 and S4.

A two-factor solution explained 84% of the variance with limited cross loading. There was substantive correlation between the factors in the rotated solution (r = 0.56), supporting the use of oblique rotation which permits factors to be correlated. Eigenvalues for each of the initial 31 factors and factor loadings (pattern and structure matrix) for each gene are presented in supplementary Tables S3 and S4. *ISG15* loaded most strongly onto Factor 1; *TAP1* loaded most strongly onto Factor 2; *CASP1* did not substantively load onto either factor. Having excluded *CASP1*, *IFI6*, *HERC5*, *EIF2AK2* & *MX1* were cross-loaded. Table 1 shows the genes that contributed to each factor; we called these IFN Score A and IFN Score B. The relationship between IFN-Score A and B elements and biological functions obtained from GO; Geneontology and KEGG; Kyoto Encyclopedia of Genes and Genomes Databases is shown in supplementary Figs S2, S3 and S4 and Tables S7, S8 and S9.

**Table 1:** Gene expression Interferon Scores A and B.

Gene	Reference from literature		Module			Rotated factor loading	
	Subsets	PB	1.2	3.4	5.12	Factor 1:IFN Score-A	Factor 2: IFN Score-B
*ISG15*	^16, 41, 42^	^20, 23, 24, 43^	•			0.96*	
*IFI44*	^16, 41, 42, 44^	^20, 23, 33, 34, 43, 45^	•			0.80*	
*IFI27*	^41, 42, 44^	^20, 33, 34^				0.77*	
*CXCL10*	^41, 42, 44^	^6^	•			0.71*	(−0.41)
*RSAD2*	^16, 42^	^20, 33, 43^	•			0.70*	
*IFIT1*	^41, 42, 44^	^43, 45^	•			0.67*	
*IFI44L*	^16, 41, 42^	^20, 24, 33, 43^	•			0.66*	
*CCL8*	^41^	^6^		•		0.58*	
*XAF1*	^41, 42^	^23, 24, 43, 45^	•			0.54*	
*IFI6*	^16, 41, 42^	^20, 24, 33^				0.51	0.45
*GBP1*	^41, 42^	^6^		•		0.46*	
*IRF7*	^16, 41, 42^	^43^		•		0.46*	
*CEACAM1*	^42^	^6^		•		0.45*	
*HERC5*	^16, 41, 42^	^20^	•			0.43	0.59
*EIF2AK2*	^16, 41, 42^	^45^		•		0.42	0.64
*MX1*	^16, 41, 42^	^20, 23, 24, 43^	•			0.40	0.56
*LAMP3*	^41, 42^	^43^	•				0.40*
*IFIH1*	^41, 42^	^43^		•			0.45*
*PHF11*	^41^	^6^			•		0.58*
*SERPING1*	^41, 42^	^24^	•				0.60*
*IFI16*	^41, 42, 44^	^6^			•		0.64*
*BST2*	^41, 42, 44^	^6, 43^			•		0.74*
*SP100*	^42, 44^	^6^			•		0.74*
*NT5C3B*		^6^			•		0.80*
*SOCS1*	^41, 42^	^6^		•			0.84*
*TRIM38*	^41^	^6^			•		0.87*
*UNC93B1*	^41^	^6^			•		0.88*
*UBE2L6*	^16, 42^	^43^		•			0.89*
*STAT1*	^16, 41, 44^	^6^		•			0.94*
*TAP1*	^16, 44^	^43^			•		0.98*
*CASP1*	^41, 42^	^6^			•	<0.40	<0.40

Rotated factor loadings show the results of factor analysis. Values are substantive factor loadings (promax; pattern matrix exceeding 0.4) after rotation. *Indicates genes that were included in the factor scores. See supplementary material for further details of factor analysis.

### Gene expression scores differ between cell subsets

**Figure 1:**
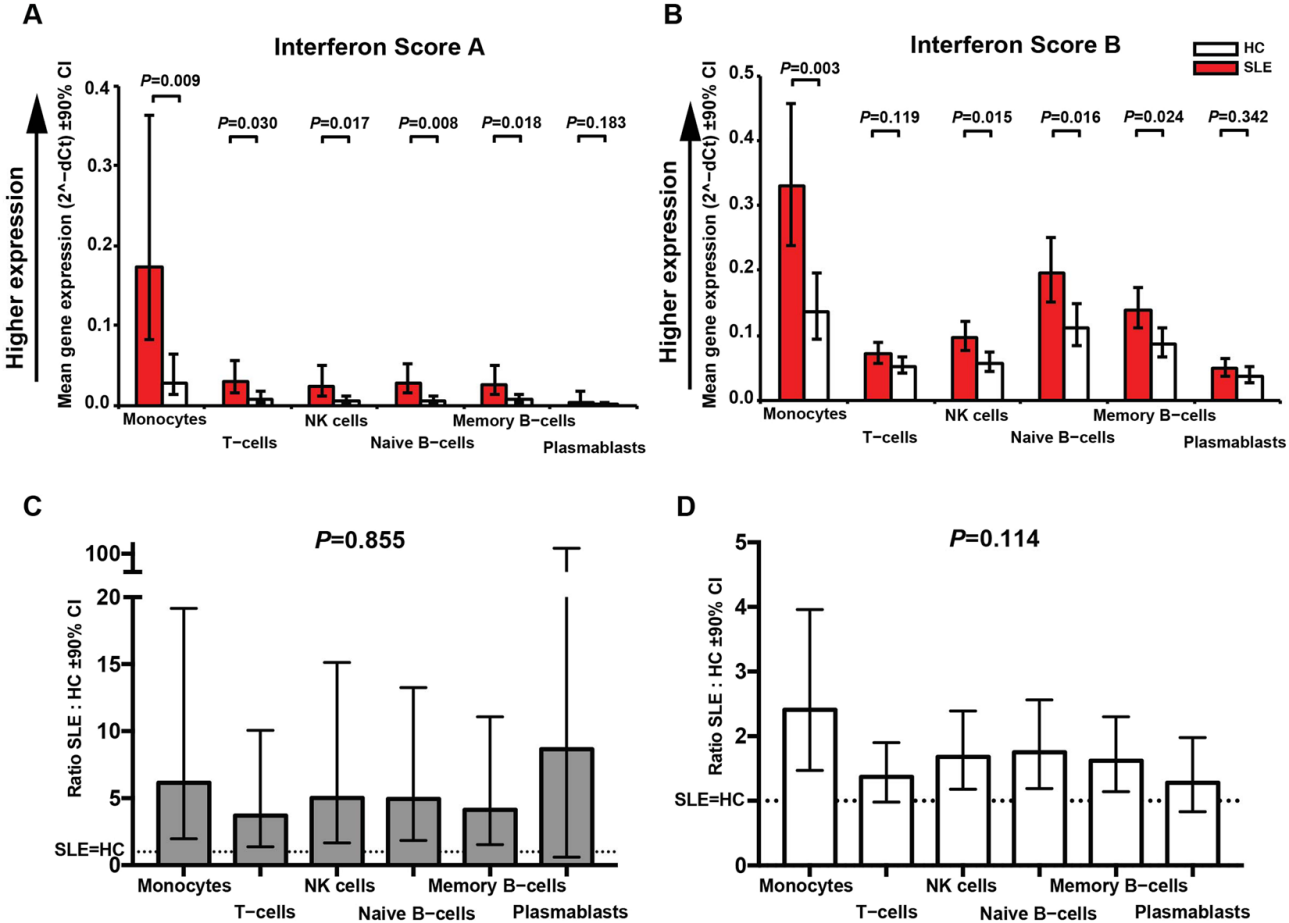
Comparison of expression of IFN Score A and IFN Score B between sorted immune cell subsets Age-adjusted level of expression (2^-dCT^) of IFN Score A (Fig. 1A) and IFN Score B (Fig. 1B) in patients with SLE (red) and HC (white) for each cell subsets identified and sorted by flow cytometry of PBMCs. Figures 1C and D show ratio of expression (SLE:HC) for IFN Score A and Score-B respectively. A substantive increase in expression was observed for both scores in each subset. For both scores expression was greatest in monocytes. Dotted lines represent SLE = HC; details in Table S4.

We sorted cells to compare the contribution of each immune cell subset to these Scores. PBMCs from 10 SLE patients and 8 HC were sorted into CD14^+^ monocytes, CD3^+^ T cells and CD56^+^CD3^−^ NK cells, CD19^+^CD27^−^CD38^−^ naïve B cells, CD19^+^CD27^+^CD38^−^ memory B cells, and CD19^+^CD27^+^CD38^+^ plasmablasts. Overall levels of expression are shown in Fig. 1 and full data with statistical tests are shown in Table S5.

In SLE patients, expression of IFN Score A was significantly higher in monocytes than in other cell subsets. While scores were lower in HC, they again reflected higher expression in monocytes relative to other cell subtypes. We next compared SLE and healthy control for each subset (between-group ratio, Fig. 1, Table S5). Expression levels in SLE patients exceeded those in HC to varying degrees depending on the subset.

The SLE:HC ratio for each subset was compared with monocytes (Table S5). For IFN Score A, there was not a statistically significant difference between monocytes and most cell subsets, although the ratios were substantively different. (Χ^2^_(5)_ = 2.0, *P* = 0.855). The trends differed somewhat for IFN Score B. In SLE patients, expression levels were higher in monocytes than in all other cell subsets, as was found for IFN Score A. The largest differences between SLE and HC were seen in monocytes (2.4-fold elevation in SLE). However, in the remaining subsets the between-group differences were considerably smaller. At the 10% level of significance, the between group difference was 43% (*P* = 0.023), 33% (*P* = 0.067) and 47% (*P* = 0.004) smaller for T cells, memory B cells and plasmablasts respectively compared to monocytes; at the 20% level of significance there was evidence suggestive of overall variation between cell subtypes in the SLE:HC IFN Score B expression ratio (Χ^2^_(5)_ = 8.9, *P* = 0.114).

### Interferon Stimulated Gene Expression following *in-vitro* stimulation

**Figure 2:**
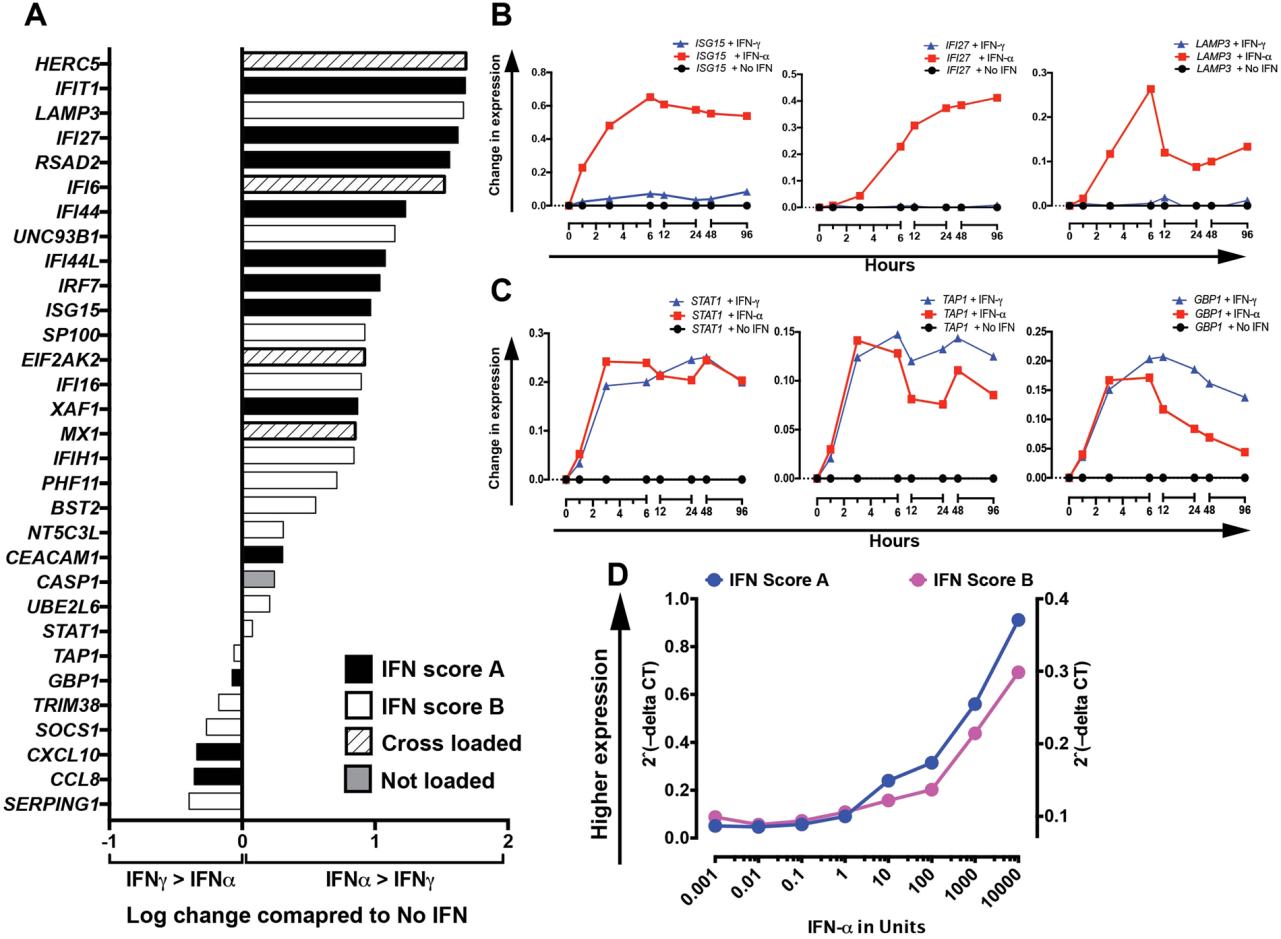
*In vitro* stimulation and expression of ISGs Expression of selected ISGs was measured following *in vitro* stimulation of B cells using either IFN-α or IFN-γ. Activated B cells were exposed to media alone or IFN-α or IFN-γ for between 1 and 96 hours before analysis of gene expression profile. (**A**) Shows log of ratio of increase in expression 6 hours after IFN-α vs. IFN-γ each compared to no stimulation. Results are mean of 3 healthy donors. Values greater than 0 indicate greater increase in expression with IFN-α than IFN-γ. Values below 0 indicate greater increase in expression with IFN-γ than IFN-α. (**B**) Shows change in expression of 3 ISGs that predominantly respond to IFN-α. (**C**) Shows change in expression for 3 ISGs, which do not demonstrate selective response to IFN-α in B cells. (**D**) For dose-response, unsorted PBMCs were stimulated with increasing doses of IFN-α. RNA was extracted from unsorted PBMCs and used to measure expression of Interferon Score-A and Interferon Score-B.

The influence of IFN-α (IFN-I) and IFN-γ (IFN-II) on B cells were compared by adding each to a B cell culture system as previously described^29^. Hierarchically clustered heat map representation of global gene expression profiles showed that many ISGs were responsive to both IFN-α and IFN-γ, while other ISGs responded specifically to IFN-α^29^. We analysed data for the 31 genes that we had selected for qPCR analysis in the present study. Fold change was measured at 6 hours as this time point showed the greatest and most consistent change across all genes and both types of IFN. Fold change for each IFN compared to media alone was calculated. The ratio of fold change between IFN-α and IFN-γ is shown in Fig. 2A. For most genes, IFN-α fold change exceeded IFN-γ. For 7 of the 31 genes this was not found. Data for all time-points is shown for 3 genes that responded predominantly to IFN-α (Fig. 2B), and 3 genes that responded to both IFN-α and IFN-γ (Fig. 2C). Both scores contained a number of genes whose expression was more responsive to IFN-α allowing the possibility that non-IFN pathways may contribute to the clustering of ISGs found between modules / factors.

**Figure 3:**
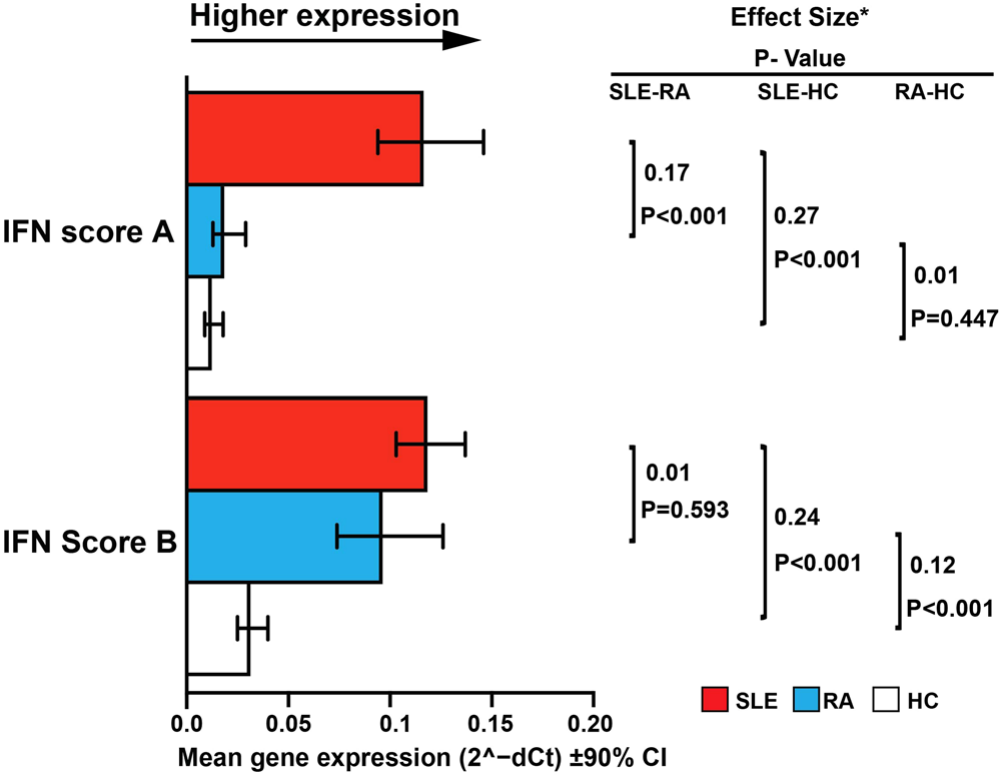
Comparison of gene expression IFN scores against diagnosis. Age-adjusted differences between patients with SLE (red) and patients with active RA (DAS28 > 3.2; blue) or HC (white) in (A) IFN Scores. Effect sizes (partial eta squared) indicate which of the variables differed to the greatest extent between the different groups. We considered effect size 0.01 to be small, 0.06 to be medium and 0.14 to be large^40^.

We next tested whether a dose-response relationship to IFN-α was present for both scores. We stimulated PBMCs with doses of IFN-α from 0.01–10,000 U/mL. TaqMan gene expression were performed after 48 hours (Fig. 2D). For gene expression, a dose dependent increase was observed between 1 and 1000 units of IFN-α. For gene expression on unsorted PBMCs, dose response was similar using IFN Score A or IFN Score B.

### Clinical validation of IFN assays: diagnostic groups

We compared the performance of the ISG expression scores in distinguishing between patients with SLE (SLICC 2012 criteria, n = 114) or RA (ACR-EULAR 2011 criteria, n = 32) versus HC (n = 49), controlling for age (Fig. 3), using ratio of expression and effect size. Full statistical table is shown in Table S6.

Comparing SLE with HC, we found the largest ratio and effect size for IFN Score A with ratio 9.35 (5.50, 15.90). Although the ratio was smaller [3.90 (2.76, 5.52)] for IFN Score B, the effect size was very similar. When comparing SLE with RA, IFN Score A was elevated in SLE [ratio 6.11 (3.42, 10.89); large effect size]; IFN Score B did not differ greatly between SLE and RA [ratio 1.26 (0.87, 1.85)]. However, when comparing RA to HC, the reverse was true; IFN Score A did not differ [ratio 1.53 (0.75, 3.14), no detectable effect], whilst IFN Score B was elevated in RA patients [ratio 3.09 (1.93, 4.94), medium-large effect size]. To summarise: compared to HC, IFN Score A was only increased in SLE, while IFN Score B was increased in both SLE and RA.

### Clinical validation of IFN scores: disease activity

**Figure 4:**
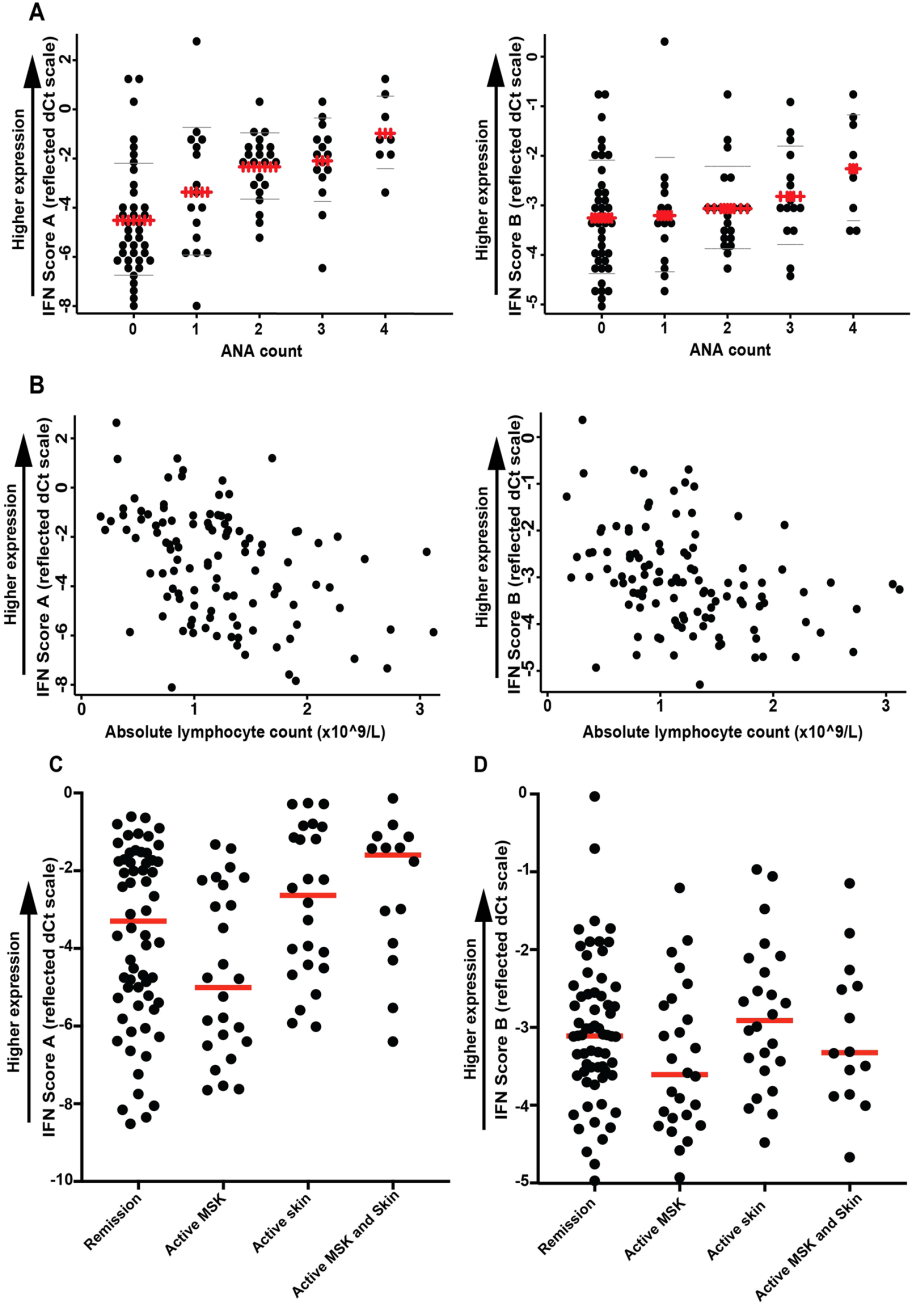
Association of IFN scores with other clinical immunology parameters Associations between each IFN score with (**A**) the number of ANA present, and (**B**) absolute lymphocyte count (observed data only). In panel A, ‘ + ’ = mean, ‘−‘ = mean ± 1 SD. ANA count refers to number of anti-extractable nuclear antigen (ENA) antibodies; including: Anti- Sm, RNP, Ro (SS-A), La (SS-B), Jo-1, Scl-70. (**C**) Shows Score A for patients with no disease activity (n = 66), active disease in the mucocutaneous system only (BILAG A or B, n = 24), active disease in the musculoskeletal system only (BILAG A or B, n = 26), or activity in both systems at the same time (n = 14). (**D**) Shows IFN Score B for the same patient groups.

In 114 SLE patients, imputation was required to address missing data in the BILAG scores (n = 2 patients), absolute lymphocyte count (n = 2), low complement (n = 9), ANA count (n = 14) and anti-dsDNA antibody titre (n = 20). IFN Score A was associated with ANA count (ratio per additional ANA reactivity (90% CI) 1.52 (1.23, 1.88), effect size = 0.13, *P* < 0.001; Fig. 4A) and lower lymphocyte count (ratio per 10^9^/L 0.47 (0.30, 0.73) effect size = 0.10, *P* = 0.001; Fig. 4B), but not age, gender, complement or anti-dsDNA antibody titre (all effect size < 0.01, *P* > 0.100). IFN Score B was not associated with ANA count (1.07 (0.95, 1.22), effect size = 0.01, *P* = 0.266), but the negative association with lymphocyte count remained substantive (0.66 (0.52, 0.83), effect size = 0.10, *P* = 0.001).

We used each IFN score as the outcome in multivariable linear regression models that included indicators for presence of activity (A, B or C) in the three most common BILAG domains (mucocutaneous, musculoskeletal and haematological, active in 46, 59 and 57 patients respectively) as independent variables, controlling for age and the blood markers of disease activity (excluding lymphocyte count since this is a component of the BILAG haematological score). This revealed a positive association between IFN Score A expression and mucocutaneous activity [ratio (90% CI) 1.71 (1.02, 2.86), effect size = 0.04, *P* = 0.042] and haematological activity [1.90 (1.11, 3.24), effect size = 0.04, *P* = 0.020], but not musculoskeletal activity [0.79 (0.46, 1.34), effect size = 0.00, *P* = 0.381]. Of the covariates, only ANA count was associated with IFN Score A expression [ratio per additional ANA 1.57 (1.26, 1.95), effect size = 0.15, *P* < 0.001]. The same pattern was seen for IFN Score B [1.41 (1.08, 1.84), effect size = 0.05, *P* = 0.012; haematological 1.44 (1.09, 1.90), effect size = 0.05, *P* = 0.012; musculoskeletal 0.82 (0.63, 1.08), effect size = 0.01, *P* = 0.165]. This variation between organ systems may explain the lack of association with total BILAG (IFN Score A *P* = 0.906, Score-B *P* = 0.780). In Fig. 4C and D we present data to illustrate the relationship between skin and musculoskeletal disease activity and IFN scores. Numbers of patients with involvement of other organs were not sufficient for multivariate anlysis.

In the longitudinal casenote review, 59/60 the patients selected had complete data. We found a significant association between IFN cores and other clinical features of disease. Notably, a different relationship was found for each score and each disease feature. Objective flare occurred in 18/59 patients and was associated with significantly higher level of IFN Score A but not Score B. Median (IQR) Score A was 0.39 (1.08) for patients with flare compared to 0.024 (0.029) for patients without (p = 0.042). IFN Score B values were 0.34 (0.50) and 0.17 (0.30) respectively (p = 0.343). In contrast, internal organ involvement was present in 21/59 patients and was associated with a trend to higher levels of IFN Score A but significantly higher IFN Score B. Median (IQR) Score A was 0.04 (1.15) for patients with internal organ involvement compared to 0.02 (0.40) for patients without (p = 0.124). IFN Score B values were 0.28 (0.64) and 0.12 (0.33) respectively (p = 0.037).

### Bimodality

To explore bimodality, we created finite mixture models on IFN Score A and IFN Score B to determine whether they represented a mixture of 2 or more distributions. We also investigated individual genes for mixture distributions.

**Figure 5:**
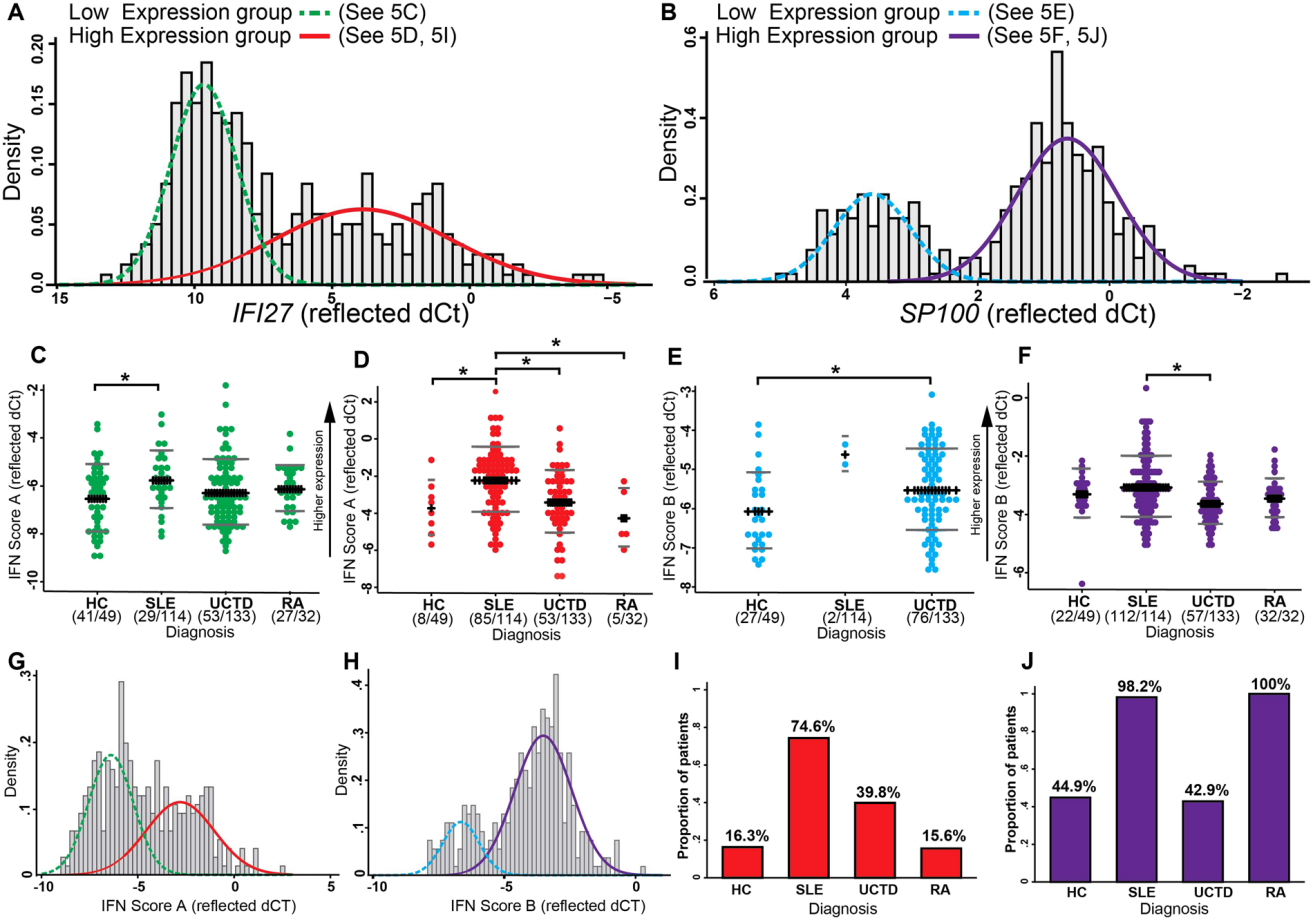
Bimodality of IFN score genes. Analysis of bimodality was performed using samples from all SLE, RA, UCTD and HC individuals (n = 328). A, B, G, H show histograms of reflected dCt values overlaid with estimated density functions for 2 separate normal distributions (solid line = ‘high’ expression; dashed line = ‘low’ expression) identified using finite mixture modelling of (**A**) IFI27; (**B**) SP100; (**G**) Score A; (**H**) Score B. In common with previous studies we observed a bimodal distribution of IFN activity as measured by each IFN score (G and H). We used IFI27 (**A**) and SP100 (**B**) as the most bimodal gene within Score A and B respectively to classify patients into low and high expression groups. (**C**–**F**) Show the level of IFN Score (reflected dCT) for each group of individuals within the high and low expression groups. These results show that SLE patients in the high expression group had a significantly higher level of Score A than RA and UCTD patients or HC individuals in the high expression group (*P < 0.1). (**I**) and (**J**) show the proportions of individuals classified within the high expression group for each diagnosis; notably 16.3% and 44.9% of HC were classified within the high group.

IFN Score A genes found to bimodally distributed were: *IFI27, ISG15, CEACAM1, IFI44L, IFI44*. The most strongly bimodal of these was *IFI27*; assigning patients to a ‘high’ or ‘low’ expression group based on this single gene instead of IFN Score A (Fig. 5A) increased the proportion with high expression from 45% to 46% and improved the mean probability of group membership from 0.89 to 0.92. IFN Score B genes found to be bimodally distributed were: *SP100, NT5C3B, UNC93B1, TRIM38, STAT1, TAP1, BST2, UBE2L6, PHF11 & IFI16*. The most strongly bimodal of these was *SP100*. Classifying patients using *SP100* alone compared to IFN Score B (Fig. 5B) increased the proportion with high expression from 20% to 32% and improved mean probability of group membership from 0.97 to 0.99.

Mean IFN scores according to expression groups are plotted in Fig. 5C–F. Interestingly, high expression group of IFN Score-A was observed in most SLE patients (85/114) but not in the other groups (HC 8/49; UCTD 53/133; RA 5/32). In contrast, high expression of IFN Score B was seen in almost all SLE (112/114) and all RA patients (32) as well as many UCTD (57/133). Notably, 22/49 HC were also in the high group.

Overall, both IFN scores were found to be from a mixture of 2 distributions with a degree of overlap (Fig. 5G,H). The proportions of individuals classified as having high expression on *IFI27* (IFN Score A) or *SP100* (IFN Score B), according to diagnosis, are presented in Fig. 5I,J.

Importantly, within each expression group, IFN scores still discriminated between diagnoses; Adjusting for age, in the high expression group SLE showed significantly higher IFN Score A than each other diagnosis. In the low expression group, IFN Score A differed between SLE and HC. For IFN Score B, in the high expression group, SLE showed higher expression than UCTD. Patients with UCTD in the low expression group had higher IFN Score B than HC.

In summary, although each IFN Score is bimodally distributed, analyzing the score as a continuous variable is more informative than by a simple high or low classification.

## Discussion

In this study, we present a novel approach to measure IFN activity in autoimmunity. We developed and clinically validate a continuous two-score system, as well as its biological basis.

Previous microarray data demonstrated co-clustering ISGs in modules to decipher multiple IFN signatures within SLE patients. These modules revealed different strength of association with disease activity. In a previous publication expression of these modules in patients receiving exogenous IFN-α and IFN-β for hepatitis C and multiple sclerosis respectively suggested that module M1.2 was responsive only to IFN-α and modules M3.4 and M5.12 were also responsive to IFN-β^6^. These data may have been affected by the disease under study, but interferome data also indicated that modules M1.2 and M3.4 were induced by IFN-β more than IFN-α and both M3.4 and M5.12 were also responsive to IFN-II (IFN-γ)^6^. Our factor analysis segregates ISGs in a similar fashion to this work: Score B mostly included genes found in M3.4 and M5.12. However, although there was a trend to a less selective IFN-α response in Score B our *in vitro* data did not show such a clear relationship between ISG subsets and subtypes of IFN.

Another hypothetical explanation for these ISG sets is that they reflect changes in numbers of circulating immune cells, which characteristically occur with disease activity in autoimmunity or immunosuppressive therapy, such as lymphopenia^16^. Our results did not support this explanation; we found both scores to be upregulated in SLE in all cell types. We cannot exclude the possibility that other non-IFN immune mediators may influence the clustering of ISGs. Further studies would be needed to answer this. Nevertheless, we have shown that separating ISG expression into two scores is clinically relevant. Most notably, many papers have described IFN activity in RA^3,4^, but we show that this is qualitatively different to SLE, with elevation of IFN Score B only.

Our factor analysis also expanded on previous work by measuring IFN activity as a continuous score instead of the bimodal classification as “IFN-high” and “IFN-low” commonly reported in other studies. Previous studies defined variable IFN-scores using as bimodal (High/low) [5, 6] either by 95% centile of Healthy donors as cutoff or >50% of the patients overlapping with HC as IFN-Low. This classification has led to inconsistent results in prediction of response to IFN-blocking therapy [6–8]. We found that both scores are indeed bimodal; there are two overlapping normal distributions underlying the distribution seen in patients (Fig. 5A and B). However, these distributions are not equivalent to normal and abnormal; many healthy individuals were in the high group. Further, there was variation within the high and low groups according to diagnosis. It therefore appears more appropriate to use a continuous measure of IFN activity in research or clinical practice.

Other ISG-based assays showed association with disease activity in cross-sectional studies^23,24,30^, particularly with skin lupus, but not with other common manifestations such as arthritis and haematological disease^15^. However, these were inconsistent with other studies failing to demonstrate any association^11,13^. Most did not adjust for activity in multiple organs and confounders. This is important because of the clinical and immunological heterogeneity of SLE. Patients with active or inactive disease in one organ system may have simultaneous activity in other organ systems independently. We controlled for this variability with multivariate analysis and by comparing patients with single-organ disease only. This revealed a variable relationship between IFN activity and different manifestations of SLE, with notably increased IFN activity in skin, but not musculoskeletal, disease activity. This explains the poor or inconsistent relationship between IFN and global disease activity reported in other studies. Both of our IFN scores showed associations with serology and features of disease activity (BILAG domains) in adjusted models, albeit with some differences in strength, indicating their advantage for stratifying disease. Further, we found a difference in interferon profile between patients who experienced flares and patients with internal organ involvement, which suggests that these two scores will be of greater value in longitudinal studies, which we are currently conducting.

In conclusion, we describe novel IFN scores as a new method to analyse the IFN-I pathway in autoimmunity. These scores should be used to measure IFN status in stratification research. Our future work will further assess the clinical usefulness of IFN-I response assays, including prediction of clinical progression in at risk individuals, flares, and response to conventional and B cell-targeted therapy.

## Methods

### Patients and healthy donor participants

All individuals gave informed written consent and research was carried out in compliance with the Helsinki Declaration. The patients’ blood samples used for this study were collected under ethical approval, REC 10/H1306/88, National Research Ethics Committee Yorkshire and Humber–Leeds East, and healthy control participants’ peripheral blood was collected under the study number 04/Q1206/107. All experiments were performed in accordance with relevant guidelines and regulations.

SLE patients met ACR/SLICC 2012 criteria. All RA patients met ACR/EULAR 2010 criteria (ACPA-positive but ANA-negative)^31^. Disease activity was assessed using BILAG-2004^32^. Undifferentiated connective tissue disease (UCTD) patients were individuals with positive ANA but did not meet criteria for CTD. Healthy controls (HC) had no autoimmune diseases or other infectious or inflammatory disease at the time of sampling. Absolute lymphocyte count was obtained from a routine diagnostic laboratory and used to calculate absolute counts for flow cytometry subsets. Number of reactivates against dsDNA, Ro52, Ro60, La, Sm, RNP, Sm/RNP and chromatin was measured using Bioplex 2200 and expressed as “ANA count”.

### Flow cytometry and cell sorting

Peripheral blood mononuclear cells (PBMCs) were separated using density gradient method (Lymphoprep^TM^, Alere Technologies, Norway) from EDTA-anticoagulated blood. Flow cytometry was performed using a Becton Dickinson (BD) LSRII flow cytometer and analysis using BD FACSDiva software. A six-way FACS was performed on a BD Influx™ to pure lymphocyte subsets. Purity of sorted populations was confirmed before RNA extraction using flow cytometry (>98% purity for each subset) as well as expression of lineage marker genes. Antibodies are shown in Table S1.

### Gene probe selection and gene expression

10 genes were selected from each IFN-annotated module (M1.2, M3.4, M5.12)^15^, with additional common ISGs (*IFI27*, *IFI6)*^20,24,33,34^. The selected genes were validated by meta-analysis of multiple GEO data biosets^35^ comparing PBMCs from SLE versus HC on Nextbio web engine^36^. Peptidylprolyl isomerase A (cyclophilin A) (*PPIA*) was used as a reference gene (confirmed not to respond to IFN-I^37^).

### Gene expression studies

Total RNA purification kit (Norgen Biotek, Canada) was used to extract RNA from PBMCs and sorted cell subsets. For cDNA synthesis from total RNA acquired, Fluidigm^®^ Reverse Transcription Master Mix buffer was used according to manufacturer’s instructions including a mixture of random primers and oligo dT for priming. TaqMan assays (Applied Biosystems, Invitrogen) were used to perform the qPCR (Table S2). TaqMan Gene Expression Assays were performed using the BioMark™ HD System and appropriate cycling protocols for the 96.96 chip. Expression of lineage markers and ISGs was analysed by relative quantitative reverse transcription–polymerase chain reaction (qRT–PCR) using TaqMan reagents from Applied Biosystems. Gene expression data were normalised to *PPIA* and calculated using ΔCt method for analysis, then transformed using 2^−Δ (Δ)Ct^.

### Multiple Imputation

Predictive mean matching (using 10 nearest neighbours) was used for continuous variables, logistic regression for binary variables. Models included age and IFN scores. We combined results from analyses performed in 20 imputed datasets according to Rubin’s rules. Effect sizes from each imputed dataset were Fisher z transformed, averaged across imputations, then back-transformed.

### Factor Analysis

Factor analysis (FA) was used to determine whether the gene expression values of multiple genes were driven by a smaller number of unobserved (latent) continuous variables. FA included expression levels for ISGs measured in the PMBCs from SLE, UCTD, RA and HC.

Prior to factor analysis (FA), undetected ∆Ct values were singly imputed using the R package nondetects^38^. The Kaiser-Meyer-Olkin measure was used to verify the sampling adequacy of the analysis^39^. Principal factor extraction, without rotation, was used to identify the optimum number of factors, which was initially determined according to a parallel analysis (Monte Carlo simulation using 1000 replications). This indicated the maximum number of factors present, but if a smaller number of factors were required to explain 80% of the variance, and resulted in lower levels of cross-loading (genes loaded by 2 or more factors at >0.4), a simpler structure was selected. Having identified the number of factors present, oblique (promax; kappa = 4) rotation was used to obtain the final factor solution. To calculate factor scores, within each patient median gene expression was calculated for genes loaded at ≥0.4 by each factor, provided they did not cross-load onto more than one factor. The advantage of this approach was that it reflected the variability of the data, and respected the within-patient ordinal scaling of delta Ct values where some genes were below the detection threshold, but yielded factor scores in units (delta Ct) that were easily interpreted in subsequent analyses.

Factor scores were calculated as the median expression (dCt scale) of genes loaded by each factor at ≥0.4, provided they were not loaded by more than one factor.

### Statistical Analysis

To compare gene expression levels between sorted cell subtypes, substantive descriptive differences were considered evidence of heterogeneity, given the sample size. We considered *P* < 0.1 indicative of potential differences, and *P* < 0.2 indicative of potential interactions.

Multilevel linear regression modeling (random intercepts, fixed slopes, unstructured covariance) was used to assess whether a) expression levels differed between sorted cell subtypes in SLE patients and b) the differences between SLE patients and HC differed according to subset type, controlling for age. Cell subsets were considered nested at the patient level. Monocytes were the reference against which other cell types were compared.

Analysis of covariance, controlling for age, compared patients with SLE to those with RA and to HC. Post-hoc pairwise tests were corrected for multiplicity (Tukey HSD method). Effect sizes (partial omega squared) were calculated; we considered effect sizes to be small (0.01), medium (0.06) or large (0.14) as described by Cohen^40^.

Multiple imputation by chained equations was used to impute missing disease activity measures within SLE patients (for details see supplementary material).

IFN scores were modelled as a function of ANA count, low complement, lymphocyte count and anti-dsDNA titre using linear regression. Linear regression was then used to relate IFN scores to presence of individual BILAG organ activity (A, B or C) controlling for age and each of the blood measures of disease activity described above. Quantile (median) regression was used to relate total BILAG and SLEDAI scores to IFN scores, controlling for the same covariates.

Finite mixture modelling (FMM) was used to explore whether IFN-Scores-A and -B each represented a mixture of 2 or more distributions. On the dCt scale, normal (Gaussian) distributions were assumed. Akaike’s Information Criterion (AIC) and Bayesian Information Criterion (BIC) were used to compare the fit of models with 1, 2 or more components. We restricted the analysis of distribution of individual gene expression to patients in whom expression could be measured.

We performed an analysis of available longitudinal data in our patients as a pilot for future formal longitudinal studies. We selected 60 patients across a spectrum of levels of IFN Score A and Score B and reviewed casenotes for objective flare and involvement of internal organs. Objective flare was defined by documentation of flare accompanied by clinical signs such as joint swelling, or confirmatory investigations such as renal biopsy, as well as new or increased treatment within 3 months of biomarker testing. Internal organ involvement was defined by confirmed and treated lupus activity in cardiorespiratory, renal and CNS systems at any prior date. 2^−dCT^ values were compared using Mann Whitney U tests.

## Electronic supplementary material

Supplementary material, methods, figures and tables

## References

[1] Deng Y, Tsao BP (2010). Genetic susceptibility to systemic lupus erythematosus in the genomic era. Nature reviews. Rheumatology.

[2] Psarras, A., Emery, P. & Vital, E. M. Type I interferon-mediated autoimmune diseases: pathogenesis, diagnosis and targeted therapy. *Rheumatology (Oxford)*, 10.1093/rheumatology/kew431 (2017). 10.1093/rheumatology/kew431 28122959

[3] Conigliaro P (2010). The type I IFN system in rheumatoid arthritis. Autoimmunity.

[4] de Jong TD (2016). The type I interferon signature in leukocyte subsets from peripheral blood of patients with early arthritis: a major contribution by granulocytes. Arthritis Res Ther.

[5] Chiche L, Jourde-Chiche N, Pascual V, Chaussabel D (2013). Current perspectives on systems immunology approaches to rheumatic diseases. Arthritis Rheum.

[6] Chiche L (2014). Modular transcriptional repertoire analyses of adults with systemic lupus erythematosus reveal distinct type I and type II interferon signatures. Arthritis Rheumatol.

[7] Kirou KA, Gkrouzman E (2013). Anti-interferon alpha treatment in SLE. Clin Immunol.

[8] McBride JM (2012). Safety and pharmacodynamics of rontalizumab in patients with systemic lupus erythematosus: results of a phase I, placebo-controlled, double-blind, dose-escalation study. Arthritis Rheum.

[9] Petri M (2013). Sifalimumab, a human anti-interferon-alpha monoclonal antibody, in systemic lupus erythematosus: a phase I randomized, controlled, dose-escalation study. Arthritis Rheum.

[10] Lauwerys BR, Ducreux J, Houssiau FA (2014). Type I interferon blockade in systemic lupus erythematosus: where do we stand?. Rheumatology (Oxford).

[11] Kennedy WP (2015). Association of the interferon signature metric with serological disease manifestations but not global activity scores in multiple cohorts of patients with SLE. Lupus Sci Med.

[12] Rice GI (2013). Assessment of interferon-related biomarkers in Aicardi-Goutieres syndrome associated with mutations in TREX1, RNASEH2A, RNASEH2B, RNASEH2C, SAMHD1, and ADAR: a case-control study. Lancet Neurol.

[13] Feng X (2006). Association of increased interferon-inducible gene expression with disease activity and lupus nephritis in patients with systemic lupus erythematosus. Arthritis Rheum.

[14] Landolt-Marticorena C (2009). Lack of association between the interferon-alpha signature and longitudinal changes in disease activity in systemic lupus erythematosus. Ann Rheum Dis.

[15] Chiche L (2014). Modular Transcriptional Repertoire Analyses of Adults With Systemic Lupus Erythematosus Reveal Distinct Type I and Type II Interferon Signatures. Arthritis &amp; Rheumatology.

[16] Becker AM (2013). SLE peripheral blood B cell, T cell and myeloid cell transcriptomes display unique profiles and each subset contributes to the interferon signature. PloS one.

[17] Whitney AR (2003). Individuality and variation in gene expression patterns in human blood. Proc Natl Acad Sci USA.

[18] Higgs BW (2012). Identification of activated cytokine pathways in the blood of systemic lupus erythematosus, myositis, rheumatoid arthritis, and scleroderma patients. Int J Rheum Dis.

[19] Berry MP (2010). An interferon-inducible neutrophil-driven blood transcriptional signature in human tuberculosis. Nature.

[20] Yao Y (2009). Neutralization of interferon-alpha/beta-inducible genes and downstream effect in a phase I trial of an anti-interferon-alpha monoclonal antibody in systemic lupus erythematosus. Arthritis Rheum.

[21] Yao Yihong, Higgs Brandon W, Richman Laura, White Barbara, Jallal Bahija (2010). Use of type I interferon-inducible mRNAs as pharmacodynamic markers and potential diagnostic markers in trials with sifalimumab, an anti-IFNα antibody, in systemic lupus erythematosus. Arthritis Research & Therapy.

[22] Feng X (2015). Identification of interferon-inducible genes as diagnostic biomarker for systemic lupus erythematosus. Clin Rheumatol.

[23] Bennett L (2003). Interferon and granulopoiesis signatures in systemic lupus erythematosus blood. The Journal of experimental medicine.

[24] Baechler EC (2003). Interferon-inducible gene expression signature in peripheral blood cells of patients with severe lupus. Proc Natl Acad Sci USA.

[25] Kawasaki M (2010). Fluctuations in the gene expression of peripheral blood mononuclear cells between the active and inactive phases of systemic lupus erythematosus. Clin Exp Rheumatol.

[26] Kirou KA (2004). Coordinate overexpression of interferon-alpha-induced genes in systemic lupus erythematosus. Arthritis Rheum.

[27] Fayyaz A (2015). Haematological manifestations of lupus. Lupus Sci Med.

[28] Kaiser FH (1974). An index of factorial simplicity. Psychometrika.

[29] Care Matthew A., Stephenson Sophie J., Barnes Nicholas A., Fan Im, Zougman Alexandre, El-Sherbiny Yasser M., Vital Edward M., Westhead David R., Tooze Reuben M., Doody Gina M. (2016). Network Analysis Identifies Proinflammatory Plasma Cell Polarization for Secretion of ISG15 in Human Autoimmunity. The Journal of Immunology.

[30] Crow MK, Kirou KA, Wohlgemuth J (2003). Microarray analysis of interferon-regulated genes in SLE. Autoimmunity.

[31] Aletaha D (2010). 2010 rheumatoid arthritis classification criteria: an American College of Rheumatology/European League Against Rheumatism collaborative initiative. Ann Rheum Dis.

[32] Isenberg DA (2005). BILAG 2004. Development and initial validation of an updated version of the British Isles Lupus Assessment Group’s disease activity index for patients with systemic lupus erythematosus. Rheumatology (Oxford).

[33] Higgs BW (2011). Patients with systemic lupus erythematosus, myositis, rheumatoid arthritis and scleroderma share activation of a common type I interferon pathway. Ann Rheum Dis.

[34] Petri MA (2013). Baseline predictors of systemic lupus erythematosus flares: data from the combined placebo groups in the phase III belimumab trials. Arthritis Rheum.

[35] Barrett T (2013). NCBI GEO: archive for functional genomics data sets–update. Nucleic Acids Res.

[36] Kupershmidt Ilya, Su Qiaojuan Jane, Grewal Anoop, Sundaresh Suman, Halperin Inbal, Flynn James, Shekar Mamatha, Wang Helen, Park Jenny, Cui Wenwu, Wall Gregory D., Wisotzkey Robert, Alag Satnam, Akhtari Saeid, Ronaghi Mostafa (2010). Ontology-Based Meta-Analysis of Global Collections of High-Throughput Public Data. PLoS ONE.

[37] Riemer AB, Keskin DB, Reinherz EL (2012). Identification and validation of reference genes for expression studies in human keratinocyte cell lines treated with and without interferon-gamma - a method for qRT-PCR reference gene determination. Experimental dermatology.

[38] McCall MN, McMurray HR, Land H, Almudevar A (2014). On non-detects in qPCR data. Bioinformatics.

[39] Kaiser HF (1974). An Index of Factorial Simplicity. Psychometrika.

[40] Cohen, J. *Statistical power analysis for the behavioral sciences*. 2nd edn, (L. Erlbaum Associates, 1988).

[41] Flint SM (2016). Leucocyte subset-specific type 1 interferon signatures in SLE and other immune-mediated diseases. RMD Open.

[42] Kyogoku C (2013). Cell-specific type I IFN signatures in autoimmunity and viral infection: what makes the difference?. PloS one.

[43] Nikpour M, Dempsey AA, Urowitz MB, Gladman DD, Barnes DA (2008). Association of a gene expression profile from whole blood with disease activity in systemic lupus erythaematosus. Ann Rheum Dis.

[44] Lyons PA (2010). Novel expression signatures identified by transcriptional analysis of separated leucocyte subsets in systemic lupus erythematosus and vasculitis. Ann Rheum Dis.

[45] Kirou KA (2005). Activation of the interferon-alpha pathway identifies a subgroup of systemic lupus erythematosus patients with distinct serologic features and active disease. Arthritis Rheum.

