# Publisher Correction: A novel two-score system for interferon status segregates autoimmune diseases and correlates with clinical features

**DOI:** 10.1038/s41598-018-33062-1

**Published:** 2018-10-01

**Authors:** Y. M. El-Sherbiny, A. Psarras, M. Y. Md Yusof, E. M. A. Hensor, R. Tooze, G. Doody, A. A. A Mohamed, D. McGonagle, M. Wittmann, P. Emery, E. M. Vital

**Affiliations:** 1 National Institute of Health Research Leeds Biomedical Research Centre, Leeds Teaching Hospitals NHS Trust, Leeds, UK; 2 Experimental Haematology, Leeds Institute of Cancer and Pathology, University of Leeds, Leeds, UK; 3 Department of Rheumatology and Rehabilitation, Faculty of Medicine, Assiut University, Asyut, Egypt; 4 Clinical Pathology Department, Faculty of Medicine, Mansoura University, Mansoura, Egypt; 5 Leeds Institute of Rheumatic and Musculoskeletal Medicine, University of Leeds, Leeds, UK

Correction to: *Scientific Reports* 10.1038/s41598-018-24198-1, published online 11 April 2018

In the original version of this Article, the author M. Y. Md Yusof was incorrectly indexed. This error has now been corrected.

