# Supplementary material for: A novel two-score system for interferon status segregates autoimmune diseases and correlates with clinical features
Source: Sci Rep. 2018 Apr 11;8:5793. doi: 10.1038/s41598-018-24198-1 (PMC5895784; doi:10.1038/s41598-018-24198-1)
Supplement: Supplementary file 1 — Supplementary material, methods, figures and tables [file 41598_2018_24198_MOESM1_ESM.docx]

**A novel two-score system for interferon status segregates autoimmune diseases and correlates with clinical features**

Y.M. El-Sherbiny^1,4,5+^, A. Psarras^1,5+^, M.Y. Md Yusof^1,5^, E.M.A. Hensor^1,5^, R. Tooze^2^, G. Doody^2^, A.A.A Mohamed^1,3,5^, D. McGonagle^1,5^, M. Wittmann^1,5^, P. Emery^1,5^, E.M. Vital^1,5*^

1. National Institute of Health Research Leeds Biomedical Research Centre, Leeds Teaching Hospitals NHS Trust
2. Experimental Haematology, Leeds Institute of Cancer and Pathology, University of Leeds
3. Department of Rheumatology and Rehabilitation, Faculty of Medicine, Assiut University, Egypt
4. Clinical Pathology Department, Faculty of Medicine, Mansoura University, Egypt
5. Leeds Institute of Rheumatic and Musculoskeletal Medicine, University of Leeds

^*^Correspondence to [email: [e.m.j.vital@leeds.ac.uk](mailto:e.m.j.vital@leeds.ac.uk)]

^+^these authors contributed equally to this work

Table S1: Antibodies used for flow cytometry and cell sorting

| **Target** | **Clone** | **Manufacturer** |
| --- | --- | --- |
| CD19 | LT19 | Miltenyi Biotec |
| CD8 | BW135/80 | Miltenyi Biotec |
| CD56 | REA196 | Miltenyi Biotec |
| CD38 | REA572 | Miltenyi Biotec |
| CD27 | M-T271 | BD BioSciences |
| CD4 | VIT4 | Miltenyi Biotec |
| CD14 | TÜK4 | Miltenyi Biotec |
| CD69 | FN50 | BD BioSciences |

## Methods

Factor analysis

When the FA was performed in the 31 genes, KMO values confirmed the sample adequacy; overall KMO=0.93 (‘superb’^[1]^) and KMO values for each of the individual genes all exceeded the acceptable 0.5 limit[^1^](#_ENREF_1). Bartlett’s test of sphericity was highly significant [Χ^2^_(465)_=11445.2, p<0.001], indicating sufficiently large correlations between the genes to permit FA. However, The determinant of the correlation matrix was low (<0.00001) when all genes were included, indicating multicollinearity between expression values in the 31 genes. Repeating the factor analysis in a reduced set of 19 genes selected for low squared multiple correlation with the others increased the determinant to 0.00002 but gave very similar results (data not shown) therefore we proceeded with the full gene set.

The parallel analysis indicated that up to 7 factors were present in the data (Figure S1); however, a simpler solution with fewer cross-loaded items was obtained with 2 factors, which still explained 84% of the variance. Eigenvalues for each of the initial 31 factors and factor loadings (pattern and structure matrix) for each gene are presented in online supplementary tables S3 & S4.

Fig S1. Screeplot of eigenvalues produced from factor analysis

**Fig.S2 Protein-protein interaction network of ISGs loaded on IFN scores**


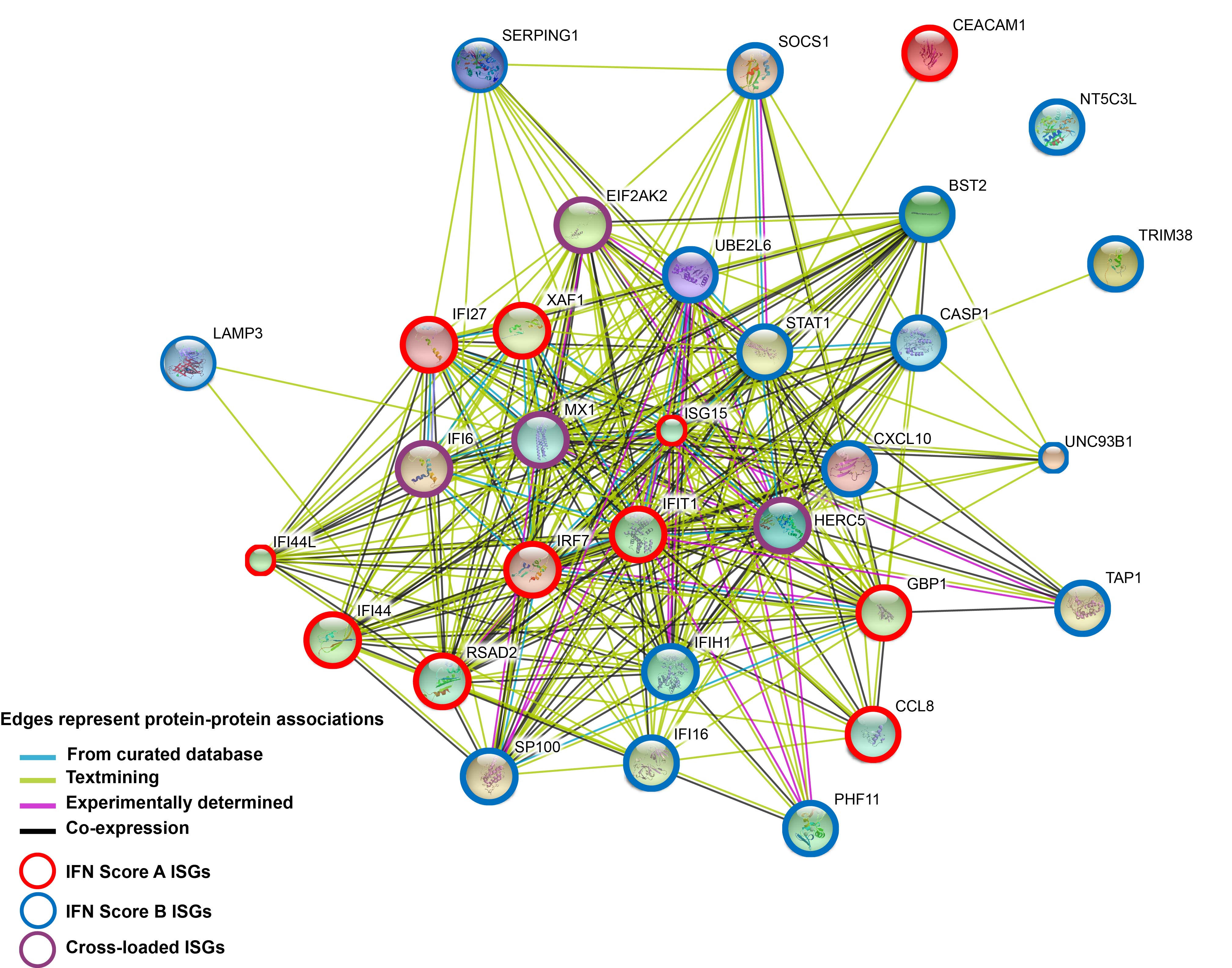


Retrieved from Interacting Gene/Proteins-database(STRING)[^2^](#_ENREF_2)

**Fig.S3 Protein-protein interaction network of ISGs loaded on IFN score A**


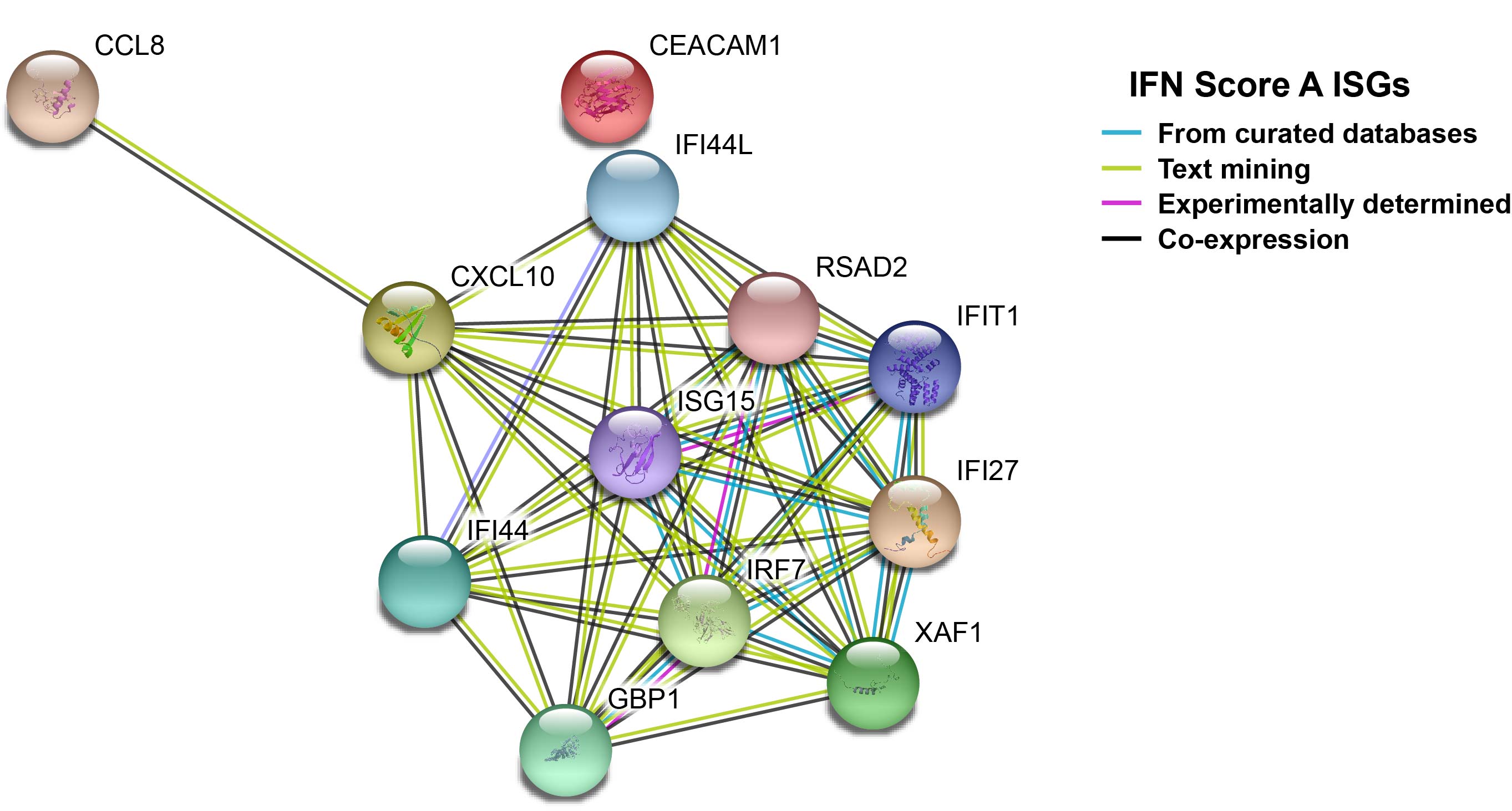


Retrieved from Interacting Gene/Proteins-database(STRING)[^2^](#_ENREF_2)

**Fig.S4 Protein-protein interaction network of ISGs loaded on IFN score B**


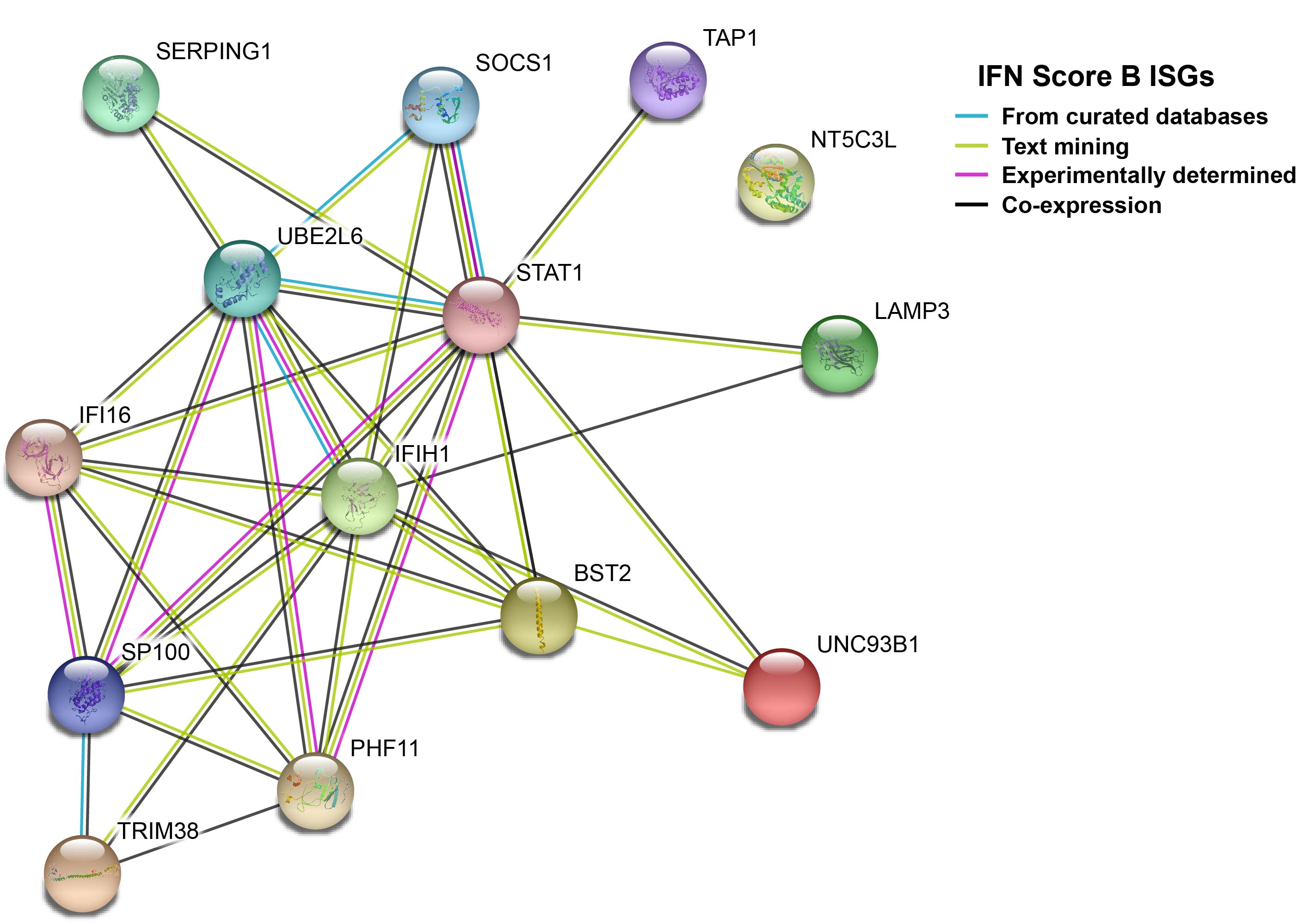


Retrieved from Interacting Gene/Proteins-database(STRING)[^2^](#_ENREF_2)

## Table S2: List of genes analysed and probe ID

| Gene | ID |  | Gene | ID |
| --- | --- | --- | --- | --- |
| AICDA | Hs00757808_m1 |  | IFNG | Hs00989291_m1 |
| BST2 A | Hs01561316_g1 |  | IGJ | Hs00376160_m1 |
| BST2 B | Hs01561315_m1 |  | IKZF2 | Hs00212361_m1 |
| CASP1 | Hs00354836_m1 |  | IL-10 | Hs00961622_m1 |
| CCL8 | Hs04187715_m1 |  | IL-10RA | Hs00155485_m1 |
| CCND2 | Hs00153380_m1 |  | IL-12A | Hs01073447_m1 |
| ITAGD | Hs00236178_m1 |  | IL-17A | Hs00174383_m1 |
| CD14 | Hs00169122_g1 |  | IL-2 | Hs00174114_m1 |
| CD163 | Hs00174705_m1 |  | IL-22 | Hs01574154_m1 |
| CD16a (FCGR3A) | Hs04188274_m1 |  | IL-28A IFNL2 | Hs00820125_g1 |
| CD19 | Hs01047410_g1 |  | IL-28B IFNL3 /2 | Hs04193047_gH |
| CD20 | Hs00544819_m1 |  | IL-28R1 IFNLR1 | Hs00417120_m1 |
| CD24 | Hs03044178_g1 |  | IL-29 IFNL1 | Hs00601677_g1 |
| IL2RA | Hs00907779_m1 |  | IL-37 | Hs00367201_m1 |
| CD27 | Hs00386811_m1 |  | IL-4 | Hs00174122_m1 |
| CD38 | Hs01120071_m1 |  | IL-6 | Hs00985639_m1 |
| CD3G | Hs00962186_m1 |  | IRF4 | Hs01056533_m1 |
| CD4 | Hs01058407_m1 |  | IRF7 | Hs01014809_g1 |
| CD69 | Hs00934033_m1 |  | ISG15 | Hs00192713_m1 |
| CD8b | Hs00174762_m1 |  | KIR3DL3 | Hs01017000_mH |
| KLRD1 (CD94) | Hs00233844_m1 |  | LAIR1 | Hs01083919_g1 |
| CD95 | Hs00236330_m1 |  | LAMP3 | Hs00180880_m1 |
| CEACAM1 | Hs00989786_m1 |  | MX1 | Hs00895608_m1 |
| CXCL10 | Hs01124251_g1 |  | MYD88 | Hs01573837_g1 |
| DNAM-1 | Hs00170832_m1 |  | NCR1 NKp46 | Hs00183118_m1 |
| EIF2AK2 | Hs00169345_m1 |  | NCR2 NKp30 | Hs00183113_m1 |
| FCER2 CD23 | Hs00233627_m1 |  | KLRK1 | Hs00183683_m1 |
| FCRL5 | Hs01070204_m1 |  | NT5C3 | Hs00369454_m1 |
| FOXP3 | Hs01085834_m1 |  | PAX5 | Hs00277134_m1 |
| GATA3 | Hs00231122_m1 |  | PHF11 | Hs00211573_m1 |
| GBP1 | Hs00977005_m1 |  | PPIA | Hs99999904_m1 |
| GUSB | Hs99999908_m1 |  | PRDM1 | Hs00153357_m1 |
| HERC5 | Hs00180943_m1 |  | RORγt | Hs01076122_m1 |
| HPRT1 | Hs99999909_m1 |  | RSAD2 | Hs00369813_m1 |
| IFI16 | Hs00194261_m1 |  | SERPING1 | Hs00163781_m1 |
| IFI27 | Hs01086373_g1 |  | SIGLEC-1 | Hs00988063_m1 |
| IFI44 | Hs00951349_m1 |  | SOCS1 | Hs00705164_s1 |
| IFI44L | Hs00915292_m1 |  | SP100 | Hs00162109_m1 |
| IFI6 | Hs00242571_m1 |  | SPATS2L | Hs01016364_m1 |
| IFIH1 | Hs01070332_m1 |  | STAT1 | Hs01013996_m1 |
| IFIT1 | Hs01911452_s1 |  | TAP1 | Hs00388675_m1 |
| IFNA1 | Hs00855471_g1 |  | TBX21 | Hs00203436_m1 |

## Table S3: Eigenvalues from factor analysis of gene expression in PBMCs

| Component | Initial Eigenvalues | | | Simulated values* |
| --- | --- | --- | --- | --- |
|  | Total | % of Variance | Cumulative % |  |
| 1 | 15.80 | 70.72 | 70.72 | 0.72 |
| 2 | 2.95 | 13.18 | 83.91 | 0.64 |
| 3 | 0.98 | 4.41 | 88.31 | 0.57 |
| 4 | 0.83 | 3.73 | 92.05 | 0.51 |
| 5 | 0.65 | 2.93 | 94.97 | 0.46 |
| 6 | 0.47 | 2.10 | 97.07 | 0.41 |
| 7 | 0.39 | 1.73 | 98.81 | 0.37 |
| 8 | 0.23 | 1.04 | 99.84 | 0.33 |
| 9 | 0.20 | 0.89 | 100.74 | 0.29 |
| 10 | 0.19 | 0.83 | 101.57 | 0.25 |
| 11 | 0.12 | 0.52 | 102.10 | 0.22 |
| 12 | 0.10 | 0.44 | 102.54 | 0.19 |
| 13 | 0.08 | 0.35 | 102.89 | 0.15 |
| 14 | 0.06 | 0.27 | 103.16 | 0.12 |
| 15 | 0.05 | 0.23 | 103.39 | 0.09 |
| 16 | 0.04 | 0.19 | 103.58 | 0.06 |
| 17 | 0.01 | 0.03 | 103.61 | 0.03 |
| 18 | 0.00 | 0.00 | 103.61 | 0.00 |
| 19 | -0.01 | -0.04 | 103.57 | -0.03 |
| 20 | -0.01 | -0.06 | 103.51 | -0.06 |
| 21 | -0.02 | -0.09 | 103.42 | -0.08 |
| 22 | -0.03 | -0.12 | 103.30 | -0.11 |
| 23 | -0.04 | -0.19 | 103.12 | -0.14 |
| 24 | -0.05 | -0.22 | 102.90 | -0.17 |
| 25 | -0.05 | -0.24 | 102.66 | -0.20 |
| 26 | -0.06 | -0.26 | 102.40 | -0.22 |
| 27 | -0.06 | -0.28 | 102.12 | -0.25 |
| 28 | -0.09 | -0.41 | 101.71 | -0.28 |
| 29 | -0.10 | -0.45 | 101.26 | -0.31 |
| 30 | -0.13 | -0.56 | 100.70 | -0.34 |
| 31 | -0.16 | -0.70 | 100.00 | -0.38 |

*Obtained via Monte Carlo simulation in 100 replications

## Table S4: Results of factor analysis of expression levels of ISG genes in PBMCs

| Gene | KMO | Pattern matrix | | Structure matrix | | Uniqueness |
| --- | --- | --- | --- | --- | --- | --- |
|  |  | Factor 1 | Factor 2 | Factor 1 | Factor 2 |  |
| BST2 | 0.94 | 0.75 | 0.20 | 0.86 | 0.62 | 0.23 |
| CASP1 | 0.87 | -0.05 | 0.34 | 0.14 | 0.32 | 0.90 |
| CCL8 | 0.89 | -0.22 | 0.59 | 0.10 | 0.46 | 0.75 |
| CEACAM1 | 0.93 | 0.18 | 0.46 | 0.44 | 0.56 | 0.66 |
| CXCL10 | 0.77 | -0.40 | 0.72 | 0.00 | 0.49 | 0.65 |
| EIFAK2 | 0.93 | 0.66 | 0.40 | 0.89 | 0.77 | 0.10 |
| GBP1 | 0.97 | 0.18 | 0.45 | 0.44 | 0.56 | 0.67 |
| HERC5 | 0.97 | 0.62 | 0.41 | 0.85 | 0.75 | 0.17 |
| IFI16 | 0.84 | 0.65 | 0.16 | 0.74 | 0.52 | 0.44 |
| IFI27 | 0.94 | 0.02 | 0.75 | 0.44 | 0.76 | 0.42 |
| IFI44 | 0.89 | 0.07 | 0.79 | 0.51 | 0.83 | 0.31 |
| IFI44L | 0.97 | 0.16 | 0.65 | 0.53 | 0.74 | 0.43 |
| IFI6 | 0.92 | 0.48 | 0.48 | 0.75 | 0.74 | 0.29 |
| IFIH1 | 0.99 | 0.47 | 0.31 | 0.64 | 0.57 | 0.52 |
| IFIT1 | 0.95 | 0.41 | 0.65 | 0.78 | 0.88 | 0.11 |
| IRF7 | 0.98 | 0.29 | 0.45 | 0.54 | 0.61 | 0.57 |
| ISG15 | 0.79 | -0.29 | 0.94 | 0.24 | 0.78 | 0.33 |
| LAMP3 | 0.96 | 0.42 | 0.34 | 0.61 | 0.57 | 0.55 |
| MX1 | 0.99 | 0.58 | 0.38 | 0.79 | 0.70 | 0.27 |
| NT5C3B | 0.90 | 0.81 | -0.34 | 0.62 | 0.11 | 0.54 |
| PHF11 | 0.91 | 0.58 | 0.27 | 0.74 | 0.60 | 0.41 |
| RSAD2 | 0.87 | 0.33 | 0.68 | 0.71 | 0.87 | 0.18 |
| SERPING1 | 0.98 | 0.62 | 0.36 | 0.82 | 0.70 | 0.24 |
| SOCS1 | 0.94 | 0.85 | -0.18 | 0.74 | 0.29 | 0.42 |
| SP100 | 0.97 | 0.74 | -0.19 | 0.63 | 0.22 | 0.57 |
| STAT1 | 0.92 | 0.95 | 0.02 | 0.96 | 0.55 | 0.08 |
| TAP1 | 0.94 | 0.99 | -0.05 | 0.96 | 0.51 | 0.07 |
| TRIM38 | 0.97 | 0.88 | -0.22 | 0.75 | 0.27 | 0.40 |
| UBE2L6 | 0.91 | 0.91 | 0.08 | 0.95 | 0.59 | 0.09 |
| UNC93B1 | 0.99 | 0.88 | -0.13 | 0.81 | 0.37 | 0.33 |
| XAF1 | 0.98 | 0.23 | 0.53 | 0.53 | 0.66 | 0.52 |

Table S5: Comparison of expression of Interferon Scores A and B between sorted immune cell subsets

|  | SLE 2^-∆Ct^  (*n*=10) | Difference in level of expression in SLE (each compared to monocytes)  2^-∆∆Ct^ (90% CI), *P* value | HC 2^-∆Ct^  (*n*=8) | Ratio in level of expression SLE:HC*  2^-∆∆Ct^ (90% CI), *P* value | Difference in SLE:HC ratio between subsets (each compared to monocytes)  2^-∆∆Ct^ (90% CI), *P* value |
| --- | --- | --- | --- | --- | --- |
| Interferon Score-A |  |  |  |  |  |
| Monocytes | 0.17 | Reference | 0.03 | 6.15 (1.97, 19.16), *P*=0.009 | Reference |
| T-cells | 0.03 | 0.17 (0.10, 0.29), *P*<0.001 | 0.01 | 3.71 (1.37, 10.06), *P*=0.030 | 0.60 (0.27, 1.34), *P*=0.300 |
| NK cells | 0.02 | 0.14 (0.10, 0.19), *P*<0.001 | 0.00 | 5.01 (1.66, 15.12), *P*=0.017 | 0.81 (0.50, 1.33), *P*=0.490 |
| Naïve B-cells | 0.03 | 0.16 (0.11, 0.24), *P*<0.001 | 0.01 | 4.95 (1.85, 13.25), *P*=0.008 | 0.81 (0.45, 1.43), *P*=0.534 |
| Memory B-cells | 0.03 | 0.15 (0.11, 0.22), *P*<0.001 | 0.01 | 4.13 (1.54, 11.07), *P*=0.018 | 0.67 (0.39, 1.16), *P*=0.230 |
| Plasmablasts | 0.00 | 0.02 (0.00, 0.07), *P*<0.001 | 0.00 | 8.65 (0.60, 124.23), *P*=0.183 | 1.41 (0.15, 13.19), *P*=0.802 |
| Interferon Score-B |  |  |  |  |  |
| Monocytes | 0.33 | Reference | 0.14 | 2.41 (1.47, 3.96), *P*=0.003 | Reference |
| T-cells | 0.07 | 0.22 (0.16, 0.28), *P*<0.001 | 0.05 | 1.37 (0.98, 1.90), *P*=0.119 | 0.57 (0.38, 0.86), *P*=0.023 |
| NK cells | 0.10 | 0.29 (0.22, 0.38), *P*<0.001 | 0.06 | 1.68 (1.18, 2.39), *P*=0.015 | 0.70 (0.46, 1.05), *P*=0.148 |
| Naïve B-cells | 0.20 | 0.59 (0.47, 0.75), *P*<0.001 | 0.11 | 1.75 (1.19, 2.56), *P*=0.016 | 0.72 (0.51, 1.02), *P*=0.125 |
| Memory B-cells | 0.14 | 0.42 (0.33, 0.53), *P*<0.001 | 0.09 | 1.62 (1.14, 2.30), *P*=0.024 | 0.67 (0.47, 0.96), *P*=0.067 |
| Plasmablasts | 0.05 | 0.15 (0.12, 0.19), *P*<0.001 | 0.04 | 1.28 (0.83, 1.98), *P*=0.342 | 0.53 (0.37, 0.77), *P*=0.004 |

Values under SLE and HC show age-adjusted mean Interferon Scores *Estimated at mean age (38.8)

## Table S6: Differences in Interferon scores between diagnosis groups

| **IFN gene expression:** | **Mean expression level (2^-∆Ct^)** | | | | **SLE-RA** | | **SLE-HC** | | | **RA-HC** | | |  |
| --- | --- | --- | --- | --- | --- | --- | --- | --- | --- | --- | --- | --- | --- |
|  | SLE (n=114) | RA (n=32) | HC (n=49) | Ratio 2^-∆∆Ct^ (90% CI), *P* value | | Effect size* | | Ratio 2^-∆∆Ct^ (90% CI), *P* value | Effect size* | | Ratio 2^-∆∆Ct^ (90% CI), *P* value | Effect size* | |
| **Interferon Score-A** | 0.117 (0.094, 0.146) | 0.019 (0.013, 0.029) | 0.013 (0.009, 0.018) | 6.062 (3.351, 10.964), *P*<0.001 | | 0.17 | | 9.263 (5.316, 16.140), *P*<0.001 | 0.27 | | 1.528 (0.743, 3.145), *P*=0.447 | 0.01 | |
| **Interferon Score-B** | 0.119 (0.103, 0.137) | 0.097 (0.074, 0.126) | 0.032 (0.025, 0.040) | 1.228 (0.835, 1.808), *P*=0.593 | | 0.01 | | 3.759 (2.617, 5.400), *P*<0.001 | 0.24 | | 3.060 (1.911, 4.900), *P*<0.001 | 0.12 | |

*Partial eta squared

All values estimated at mean age (43.2).

**Table S7: All analysed 31 ISGs elements and biological functions obtained from GO; Geneontology and KEGG; Kyoto Encyclopedia of Genes and Genomes Databases.**[**^2^**](#_ENREF_2)

| **Gene symbol** | | **Corresponding protein information** | | | | | |
| --- | --- | --- | --- | --- | --- | --- | --- |
| *CEACAM1* | | Carcinoembryonic antigen-related cell adhesion molecule 1 (biliary glycoprotein) (526 aa) | | | | | |
| *UNC93B1* | | Unc-93 homolog B1 (C. elegans); Plays an important role in innate and adaptive immunity by regulating nucleotide-sensing Toll-like receptor (TLR) signaling. Required for the transport of a subset of TLRs (including TLR3, TLR7 and TLR9) from the endoplasmic reticulum to endolysosomes where they can engage pathogen nucleotides and activate signaling cascades. May play a role in autoreactive B- cells removal (596 aa) | | | | | |
| *TRIM38* | | Tripartite motif containing 38; E3 ubiquitin-protein ligase. Mediates ’Lys-48’-linked polyubiquitination and proteasomal degradation of the critical TLR adapter TICAM1, inhibiting TLR3-mediated type I interferon signaling (465 aa) | | | | | |
| *EIF2AK2* | | Eukaryotic translation initiation factor 2-alpha kinase 2; IFN-induced dsRNA-dependent serine/threonine-protein kinase which plays a key role in the innate immune response to viral infection and is also involved in the regulation of signal transduction, apoptosis, cell proliferation and differentiation. Exerts its antiviral activity on a wide range of DNA and RNA viruses including hepatitis C virus (HCV), hepatitis B virus (HBV), measles virus (MV) and herpes simplex virus 1 (HHV-1). Inhibits viral replication via phosphorylation of the alpha subunit of eukaryotic initiation factor 2 ( [...] (551 aa) | | | | | |
| *BST2* | | Bone marrow stromal cell antigen 2; IFN-induced antiviral host restriction factor which efficiently blocks the release of diverse mammalian enveloped viruses by directly tethering nascent virions to the membranes of infected cells. Acts as a direct physical tether, holding virions to the cell membrane and linking virions to each other. The tethered virions can be internalized by endocytosis and subsequently degraded or they can remain on the cell surface. In either case, their spread as cell-free virions is restricted. Its target viruses belong to diverse families, including retrovirid [...] (180 aa) | | | | | |
| *IFIH1* | | Interferon induced with helicase C domain 1; Innate immune receptor which acts as a cytoplasmic sensor of viral nucleic acids and plays a major role in sensing viral infection and in the activation of a cascade of antiviral responses including the induction of type I interferons and proinflammatory cytokines. Its ligands include mRNA lacking 2’-O- methylation at their 5’ cap and long-dsRNA (>1 kb in length). Upon ligand binding it associates with mitochondria antiviral signaling protein (MAVS/IPS1) which activates the IKK-related kinases- TBK1 and IKBKE which phosphorylate interferon r [...] (1025 aa) | | | | | |
| *HERC5* | | HECT and RLD domain containing E3 ubiquitin protein ligase 5; Major E3 ligase for ISG15 conjugation. Acts as a positive regulator of innate antiviral response in cells induced by interferon. Functions as part of the ISGylation machinery that recognizes target proteins in a broad and relatively non-specific manner. Catalyzes ISGylation of IRF3 which results in sustained activation, it attenuates IRF3-PIN1 interaction, which antagonizes IRF3 ubiquitination and degradation, and boosts the antiviral response. Catalyzes ISGylation of influenza A viral NS1 which attenuates virulence; ISGylat [...] (1024 aa) | | | | | |
| *LAMP3* | | Lysosomal-associated membrane protein 3; May play a role in dendritic cell function and in adaptive immunity (416 aa) | | | | | |
| *SERPING1* | | Serpin peptidase inhibitor, clade G (C1 inhibitor), member 1; Activation of the C1 complex is under control of the C1- inhibitor. It forms a proteolytically inactive stoichiometric complex with the C1r or C1s proteases. May play a potentially crucial role in regulating important physiological pathways including complement activation, blood coagulation, fibrinolysis and the generation of kinins. Very efficient inhibitor of FXIIa. Inhibits chymotrypsin and kallikrein (500 aa) | | | | | |
| *UBE2L6* | | Ubiquitin-conjugating enzyme E2L 6; Catalyzes the covalent attachment of ubiquitin or ISG15 to other proteins. Functions in the E6/E6-AP-induced ubiquitination of p53/TP53. Promotes ubiquitination and subsequent proteasomal degradation of FLT3 (153 aa) | | | | | |
| *IFI27* | | Interferon, alpha-inducible protein 27; Promotes cell death. Mediates IFN-induced apoptosis characterized by a rapid and robust release of cytochrome C from the mitochondria and activation of BAX and caspases 2, 3, 6, 8 and 9 (119 aa) | | | | | |
| *CXCL10* | | Chemokine (C-X-C motif) ligand 10; Chemotactic for monocytes and T-lymphocytes. Binds to CXCR3 (98 aa) | | | | | |
| *IRF7* | | Interferon regulatory factor 7; Key transcriptional regulator of type I interferon (IFN)-dependent immune responses and plays a critical role in the innate immune response against DNA and RNA viruses. Regulates the transcription of type I IFN genes (IFN-alpha and IFN-beta) and IFN-stimulated genes (ISG) by binding to an interferon-stimulated response element (ISRE) in their promoters. Can efficiently activate both the IFN-beta (IFNB) and the IFN-alpha (IFNA) genes and mediate their induction via both the virus-activated, MyD88- independent pathway and the TLR-activated, MyD88-dependent [...] (516 aa) | | | | | |
| *SOCS1* | | Suppressor of cytokine signaling 1; SOCS family proteins form part of a classical negative feedback system that regulates cytokine signal transduction. SOCS1 is involved in negative regulation of cytokines that signal through the JAK/STAT3 pathway. Through binding to JAKs, inhibits their kinase activity. In vitro, also suppresses Tec protein- tyrosine activity. Appears to be a major regulator of signaling by interleukin 6 (IL6) and leukemia inhibitory factor (LIF). Regulates interferon-gamma mediated sensory neuron survival (By similarity). Probable substrate recognition component of a [...] (211 aa) | | | | | |
| *IFI6* | | Interferon, alpha-inducible protein 6 (138 aa) | | | | | |
| *SP100* | | SP100 nuclear antigen; Together with PML, this tumor suppressor is a major constituent of the PML bodies, a subnuclear organelle involved in a large number of physiological processes including cell growth, differentiation and apoptosis. Functions as a transcriptional coactivator of ETS1 and ETS2 according to PubMed-11909962. Under certain conditions, it may also act as a corepressor of ETS1 preventing its binding to DNA according to PubMed-15247905. Through the regulation of ETS1 it may play a role in angiogenesis, controlling endothelial cell motility and invasion. Through interaction [...] (885 aa) | | | | | |
| *TAP1* | | Transporter 1, ATP-binding cassette, sub-family B (MDR/TAP); Involved in the transport of antigens from the cytoplasm to the endoplasmic reticulum for association with MHC class I molecules. Also acts as a molecular scaffold for the final stage of MHC class I folding, namely the binding of peptide. Nascent MHC class I molecules associate with TAP via tapasin. Inhibited by the covalent attachment of herpes simplex virus ICP47 protein, which blocks the peptide-binding site of TAP. Inhibited by human cytomegalovirus US6 glycoprotein, which binds to the lumenal side of the TAP complex and [...] (808 aa) | | | | | |
| *STAT1* | | Signal transducer and activator of transcription 1, 91kDa; Signal transducer and transcription activator that mediates cellular responses to interferons (IFNs), cytokine KITLG/SCF and other cytokines and other growth factors. Following type I IFN (IFN-alpha and IFN-beta) binding to cell surface receptors, signaling via protein kinases leads to activation of Jak kinases (TYK2 and JAK1) and to tyrosine phosphorylation of STAT1 and STAT2. The phosphorylated STATs dimerize and associate with ISGF3G/IRF-9 to form a complex termed ISGF3 transcription factor, that enters the nucleus. ISGF3 bi [...] (750 aa) | | | | | |
| *XAF1* | | XIAP associated factor 1; Seems to function as a negative regulator of members of the IAP (inhibitor of apoptosis protein) family. Inhibits anti- caspase activity of BIRC4. Induces cleavage and inactivation of BIRC4 independent of caspase activation. Mediates TNF-alpha- induced apoptosis and is involved in apoptosis in trophoblast cells. May inhibit BIRC4 indirectly by activating the mitochondrial apoptosis pathway. After translocation to mitochondria, promotes translocation of BAX to mitochondria and cytochrome c release from mitochondria. Seems to promote the redistribution of BIRC4 f [...] (301 aa) | | | | | |
| *IFI16* | | Interferon, gamma-inducible protein 16; Binds double-stranded DNA. Binds preferentially to supercoiled DNA and cruciform DNA structures. Seems to be involved in transcriptional regulation. May function as a transcriptional repressor. Could have a role in the regulation of hematopoietic differentiation through activation of unknown target genes. Controls cellular proliferation by modulating the functions of cell cycle regulatory factors including p53/TP53 and the retinoblastoma protein. May be involved in TP53-mediated transcriptional activation by enhancing TP53 sequence-specific DNA b [...] (729 aa) | | | | | |
| *GBP1* | | Guanylate binding protein 1, interferon-inducible; Hydrolyzes GTP to GMP in 2 consecutive cleavage reactions. Exhibits antiviral activity against influenza virus. Promote oxidative killing and deliver antimicrobial peptides to autophagolysosomes, providing broad host protection against different pathogen classes (592 aa) | | | | | |
| *IFI44* | | Interferon-induced protein 44; This protein aggregates to form microtubular structures (444 aa) | | | | | |
| *IFI44L* | | Interferon-induced protein 44-like; Exhibits a low antiviral activity against hepatitis C virus (452 aa) | | | | | |
| *IFIT1* | | Interferon-induced protein with tetratricopeptide repeats 1; Interferon-induced antiviral RNA-binding protein that specifically binds single-stranded RNA bearing a 5’-triphosphate group (PPP-RNA), thereby acting as a sensor of viral single- stranded RNAs and inhibiting expression of viral messenger RNAs. Single-stranded PPP-RNAs, which lack 2’-O-methylation of the 5’ cap and bear a 5’-triphosphate group instead, are specific from viruses, providing a molecular signature to distinguish between self and non-self mRNAs by the host during viral infection. Directly binds PPP-RNA in a non-se [...] (478 aa) | | | | | |
| *PHF11* | | PHD finger protein 11; Positive regulator of Th1-type cytokine gene expression (331 aa) | | | | | |
| *ISG15* | | ISG15 ubiquitin-like modifier; Ubiquitin-like protein which plays a key role in the innate immune response to viral infection either via its conjugation to a target protein (ISGylation) or via its action as a free or unconjugated protein. ISGylation involves a cascade of enzymatic reactions involving E1, E2, and E3 enzymes which catalyze the conjugation of ISG15 to a lysine residue in the target protein. Its target proteins include IFIT1, MX1/MxA, PPM1B, UBE2L6, UBA7, CHMP5, CHMP2A, CHMP4B and CHMP6. Can also isgylate- EIF2AK2/PKR which results in its activation, DDX58/RIG-I which inhi [...] (165 aa) | | | | | |
| *RSAD2* | | Radical S-adenosyl methionine domain containing 2; Interferon-inducible iron-sulfur (4FE-4S) cluster- binding antiviral protein which plays a major role in the cell antiviral state induced by type I and type II interferon. Can inhibit a wide range of DNA and RNA viruses, including human cytomegalovirus (HCMV), hepatitis C virus (HCV), west Nile virus (WNV), dengue virus, sindbis virus, influenza A virus, sendai virus, vesicular stomatitis virus (VSV), and human immunodeficiency virus (HIV-1). Displays antiviral activity against influenza A virus by inhibiting the budding of the virus f [...] (361 aa) | | | | | |
| *CCL8* | | Chemokine (C-C motif) ligand 8; Chemotactic factor that attracts monocytes, lymphocytes, basophils and eosinophils. May play a role in neoplasia and inflammatory host responses. This protein can bind heparin. The processed form MCP-2(6-76) does not show monocyte chemotactic activity, but inhibits the chemotactic effect most predominantly of CCL7, and also of CCL2 and CCL5 and CCL8 (99 aa) | | | | | |
| *MX1* | | Myxovirus (influenza virus) resistance 1, interferon-inducible protein p78 (mouse); Interferon-induced dynamin-like GTPase with antiviral activity against a wide range of RNA viruses and some DNA viruses. Its target viruses include negative-stranded RNA viruses and HBV through binding and inactivation of their ribonucleocapsid. May also antagonize reoviridae and asfarviridae replication. Inhibits thogoto virus (THOV) replication by preventing the nuclear import of viral nucleocapsids. Inhibits La Crosse virus (LACV) replication by sequestering viral nucleoprotein in perinuclear complex [...] (662 aa) | | | | | |
| *NT5C3L* | | 5’-nucleotidase, cytosolic III-like; Specifically hydrolyzes 7-methylguanosine monophosphate (m(7)GMP) to 7-methylguanosine and inorganic phosphate (PubMed-23223233, PubMed-24603684). The specific activity for m(7)GMP may protect cells against undesired salvage of m(7)GMP and its incorporation into nucleic acids (PubMed-23223233). Also has weak activity for CMP (PubMed-23223233, PubMed-24603684). UMP and purine nucleotides are poor substrates (PubMed-23223233) (300 aa) | | | | | |
| *CASP1* | | Caspase 1, apoptosis-related cysteine peptidase; Thiol protease that cleaves IL-1 beta between an Asp and an Ala, releasing the mature cytokine which is involved in a variety of inflammatory processes. Important for defense against pathogens. Cleaves and activates sterol regulatory element binding proteins (SREBPs). Can also promote apoptosis (404 aa) | | | | | |
| **Biological Process (GO)** | | | | | | |  |
| **Pathway ID** | **Pathway description** | | **count in gene set** | **false discovery rate** | **Elements** | |  |
| GO:0009615 | response to virus | | 17 | 5.89E-22 | BST2, CCL8, CXCL10, EIF2AK2, GBP1, HERC5, IFI16, IFI44, IFI44L, IFIH1, IFIT1, IRF7, ISG15, MX1, RSAD2, STAT1, UNC93B1 | |  |
| GO:0051607 | defense response to virus | | 15 | 9.66E-21 | BST2,CXCL10,EIF2AK2,GBP1,HERC5,IFI16,IFI44L,IFIH1,IFIT1,IRF7,ISG15,MX1,RSAD2,STAT1,UNC93B1 | |  |
| GO:0006955 | immune response | | 24 | 1.91E-20 | BST2,CASP1,CCL8,CXCL10,EIF2AK2,GBP1,HERC5,IFI16,IFI27,IFI44L,IFI6,IFIH1,IFIT1,ISG15,MX1,RSAD2,SERPING1,SOCS1,SP100,STAT1,TRIM38,UBE2L6,UNC93B1,XAF1 | |  |
| GO:0045087 | innate immune response | | 22 | 2.18E-20 | BST2,CASP1,EIF2AK2,GBP1,HERC5,IFI16,IFI27,IFI6,IFIH1,IFIT1,IRF7,ISG15,MX1,RSAD2,SERPING1,SOCS1,SP100,STAT1,TRIM38,UBE2L6,UNC93B1,XAF1 | |  |
| GO:0006952 | defense response | | 24 | 5.60E-20 | BST2,CASP1,CCL8,EIF2AK2,GBP1,HERC5,IFI16,IFI27,IFI44L,IFI6,IFIH1,IFIT1,IRF7,ISG15,MX1,RSAD2,SERPING1,SOCS1,SP100,STAT1,TRIM38,UBE2L6,UNC93B1,XAF1 | |  |
| GO:0060337 | type I interferon signaling pathway | | 12 | 1.17E-19 | BST2,IFI27,IFI6,IFIT1,IRF7,ISG15,MX1,RSAD2,SOCS1,SP100,STAT1,XAF1 | |  |
| GO:0071357 | cellular response to type I interferon | | 12 | 1.17E-19 | BST2,IFI27,IFI6,IFIT1,IRF7,ISG15,MX1,RSAD2,SOCS1,SP100,STAT1,XAF1 | |  |
| GO:0019221 | cytokine-mediated signaling pathway | | 17 | 3.48E-19 | BST2, CXCL10, GBP1, HERC5, IFI27, IFI6, IFIT1, IRF7, ISG15, MX1, RSAD2, SOCS1, SP100, STAT1, TRIM38, UBE2L6, XAF1 | |  |
| GO:0034097 | response to cytokine | | 18 | 2.79E-17 | BST2, CXCL10, EIF2AK2, GBP1, HERC5, IFI27, IFI6, IFIT1, IRF7, ISG15, MX1, RSAD2, SOCS1, SP100, STAT1, TRIM38, UBE2L6, XAF1 | |  |
| GO:0071345 | cellular response to cytokine stimulus | | 17 | 5.79E-17 | BST2, CXCL10, GBP1, HERC5, IFI27, IFI6, IFIT1, IRF7, ISG15, MX1, RSAD2, SOCS1, SP100, STAT1, TRIM38, UBE2L6, XAF1 | |  |
| GO:0002376 | immune system process | | 24 | 6.28E-17 | BST2, CASP1, CCL8, CEACAM1, EIF2AK2, GBP1, HERC5, IFI16, IFI27, IFI44L, IFI6, IFIH1, IFIT1, ISG15, LAMP3, MX1, RSAD2, SERPING1, SOCS1, SP100, STAT1, TRIM38, UBE2L6, XAF1 | |  |
| GO:0051707 | response to other organism | | 18 | 6.28E-17 | BST2, CASP1, CCL8, EIF2AK2, GBP1, HERC5, IFI16, IFI44, IFI44L, IFIH1, IFIT1, IRF7, ISG15, MX1, RSAD2, SOCS1, STAT1, UNC93B1 | |  |
| GO:0002252 | immune effector process | | 15 | 3.97E-15 | BST2,CXCL10,EIF2AK2,GBP1,HERC5,IFI16,IFI44L,IFIH1,IFIT1,ISG15,MX1,RSAD2,SERPING1,STAT1,UNC93B1 | |  |
| GO:0048525 | negative regulation of viral process | | 9 | 1.72E-12 | BST2, EIF2AK2, IFI16, IFIT1, ISG15, MX1, RSAD2, SP100, STAT1 | |  |
| GO:0043901 | negative regulation of multi-organism process | | 10 | 4.31E-12 | BST2, EIF2AK2, IFI16, IFIT1, ISG15, MX1, RSAD2, SP100, STAT1, TRIM38 | |  |
| GO:0045069 | regulation of viral genome replication | | 8 | 4.40E-11 | BST2, EIF2AK2, IFI16, IFIT1, ISG15, MX1, RSAD2, TRIM38 | |  |
| GO:0001817 | regulation of cytokine production | | 13 | 6.01E-11 | BST2, CASP1, EIF2AK2, GBP1, HERC5, IFI16, IFIH1, IRF7, ISG15, RSAD2, SOCS1, TRIM38, UBE2L6 | |  |
| GO:1903901 | negative regulation of viral life cycle | | 8 | 1.15E-10 | BST2, EIF2AK2, IFI16, IFIT1, ISG15, MX1, RSAD2, SP100 | |  |
| GO:0006950 | response to stress | | 23 | 1.61E-10 | BST2, CCL8, CEACAM1, GBP1, HERC5, IFI16, IFI27, IFI44L, IFI6, IFIH1, IFIT1, IRF7, ISG15, MX1, RSAD2, SERPING1, SOCS1, SP100, STAT1, TRIM38, UBE2L6, UNC93B1, XAF1 | |  |
| GO:0045071 | negative regulation of viral genome replication | | 7 | 1.88E-10 | BST2, EIF2AK2, IFI16, IFIT1, ISG15, MX1, RSAD2 | |  |
| GO:0071310 | cellular response to organic substance | | 18 | 1.12E-09 | BST2, CASP1, CXCL10, EIF2AK2, GBP1, HERC5, IFI27, IFI6, IFIT1, IRF7, ISG15, MX1, RSAD2, SP100, STAT1, TRIM38, UBE2L6, XAF1 | |  |
| GO:0051704 | multi-organism process | | 19 | 1.48E-09 | BST2,CASP1,CCL8,EIF2AK2,GBP1,HERC5,IFI16,IFI44,IFI44L,IFIH1,IFIT1,IRF7,ISG15,MX1,RSAD2,SOCS1,SP100,STAT1,UNC93B1 | |  |
| GO:0002682 | regulation of immune system process | | 16 | 1.86E-09 | BST2,CCL8,CXCL10,EIF2AK2,GBP1,HERC5,IFI16,IFIH1,IFIT1,ISG15,RSAD2,SERPING1,SOCS1,STAT1,TRIM38,UNC93B1 | |  |
| GO:0070887 | cellular response to chemical stimulus | | 19 | 2.45E-09 | BST2,CASP1,CCL8,CXCL10,EIF2AK2,GBP1,HERC5,IFI27,IFI6,IFIT1,IRF7,ISG15,MX1,RSAD2,SP100,STAT1,TRIM38,UBE2L6,XAF1 | |  |
| GO:0009605 | response to external stimulus | | 17 | 1.62E-08 | BST2,CCL8,EIF2AK2,GBP1,HERC5,IFI16,IFI44,IFI44L,IFIH1,IFIT1,IRF7,ISG15,MX1,RSAD2,SOCS1,STAT1,UNC93B1 | |  |
| GO:0007165 | signal transduction | | 23 | 6.26E-08 | BST2,CASP1,CCL8,CEACAM1,CXCL10,EIF2AK2,GBP1,HERC5,IFI16,IFI27,IFI6,IFIH1,IFIT1,IRF7,ISG15,MX1,RSAD2,SOCS1,SP100,TRIM38,UBE2L6,UNC93B1,XAF1 | |  |
| GO:0007166 | cell surface receptor signaling pathway | | 17 | 6.26E-08 | BST2,CEACAM1,CXCL10,GBP1,HERC5,IFI27,IFI6,IFIT1,IRF7,ISG15,MX1,RSAD2,SOCS1,SP100,TRIM38,UBE2L6,XAF1 | |  |
| GO:0034341 | response to interferon-gamma | | 7 | 7.17E-08 | BST2,GBP1,IRF7,SOCS1,SP100,STAT1,TRIM38 | |  |
| GO:0032479 | regulation of type I interferon production | | 7 | 1.25E-07 | HERC5,IFI16,IFIH1,IRF7,ISG15,TRIM38,UBE2L6 | |  |
| GO:0060333 | interferon-gamma-mediated signaling pathway | | 6 | 1.73E-07 | GBP1,IRF7,SOCS1,SP100,STAT1,TRIM38 | |  |
| GO:0043900 | regulation of multi-organism process | | 10 | 1.92E-07 | BST2,EIF2AK2,HERC5,IFI16,IFIT1,ISG15,MX1,RSAD2,SP100,STAT1 | |  |
| GO:0044700 | single organism signaling | | 23 | 1.99E-07 | BST2,CASP1,CCL8,CEACAM1,CXCL10,EIF2AK2,GBP1,HERC5,IFI16,IFI27,IFI6,IFIH1,IFIT1,IRF7,ISG15,MX1,RSAD2,SOCS1,SP100,TRIM38,UBE2L6,UNC93B1,XAF1 | |  |
| GO:0007154 | cell communication | | 23 | 3.06E-07 | BST2,CASP1,CCL8,CEACAM1,CXCL10,EIF2AK2,GBP1,HERC5,IFI16,IFI27,IFI6,IFIH1,IFIT1,IRF7,ISG15,MX1,RSAD2,SOCS1,SP100,TRIM38,UBE2L6,UNC93B1,XAF1 | |  |
| GO:0031347 | regulation of defense response | | 11 | 8.02E-07 | CASP1,EIF2AK2,HERC5,IFI16,IFIH1,IFIT1,SERPING1,SOCS1,STAT1,TRIM38,UNC93B1 | |  |
| GO:0048519 | negative regulation of biological process | | 21 | 8.61E-07 | BST2,CCL8,CXCL10,GBP1,HERC5,IFI16,IFI27,IFI6,IFIH1,IFIT1,IRF7,ISG15,MX1,RSAD2,SERPING1,SOCS1,SP100,STAT1,TRIM38,UBE2L6,XAF1 | |  |
| GO:0071346 | cellular response to interferon-gamma | | 6 | 1.13E-06 | GBP1,IRF7,SOCS1,SP100,STAT1,TRIM38 | |  |
| GO:0032020 | ISG15-protein conjugation | | 3 | 1.93E-05 | HERC5,ISG15,UBE2L6 | |  |
| GO:0051239 | regulation of multicellular organismal process | | 15 | 3.14E-05 | BST2,CASP1,CXCL10,EIF2AK2,GBP1,HERC5,IFI16,IFIH1,IRF7,SERPING1,SOCS1,SP100,STAT1,TRIM38,UBE2L6 | |  |
| GO:0010033 | response to organic substance | | 15 | 5.65E-05 | BST2,GBP1,HERC5,IFI27,IFI6,IFIT1,IRF7,ISG15,MX1,RSAD2,SP100,STAT1,TRIM38,UBE2L6,XAF1 | |  |
| GO:0002698 | negative regulation of immune effector process | | 5 | 5.87E-05 | BST2,EIF2AK2,IFIT1,SERPING1,TRIM38 | |  |
| GO:0001818 | negative regulation of cytokine production | | 6 | 0.000122 | BST2,GBP1,HERC5,IFIH1,ISG15,UBE2L6 | |  |
| GO:0032480 | negative regulation of type I interferon production | | 4 | 0.000127 | HERC5,IFIH1,ISG15,UBE2L6 | |  |
| GO:0051241 | negative regulation of multicellular organismal process | | 10 | 0.000127 | BST2,CXCL10,GBP1,HERC5,IFIH1,ISG15,SERPING1,SP100,STAT1,UBE2L6 | |  |
| GO:0002683 | negative regulation of immune system process | | 7 | 0.000166 | BST2,EIF2AK2,GBP1,IFI16,IFIT1,SERPING1,TRIM38 | |  |
| GO:0051716 | cellular response to stimulus | | 21 | 0.000286 | BST2,CASP1,CCL8,CEACAM1,GBP1,HERC5,IFI16,IFI27,IFI6,IFIH1,IFIT1,IRF7,ISG15,MX1,RSAD2,SP100,STAT1,TRIM38,UBE2L6,UNC93B1,XAF1 | |  |
| GO:0002697 | regulation of immune effector process | | 7 | 0.000297 | BST2,EIF2AK2,HERC5,IFIT1,RSAD2,SERPING1,TRIM38 | |  |
| GO:0031348 | negative regulation of defense response | | 5 | 0.000319 | EIF2AK2,IFI16,IFIT1,SERPING1,TRIM38 | |  |
| GO:0050687 | negative regulation of defense response to virus | | 3 | 0.000421 | EIF2AK2,IFIT1,TRIM38 | |  |
| GO:0035456 | response to interferon-beta | | 3 | 0.000502 | BST2,STAT1,XAF1 | |  |
| GO:0048523 | negative regulation of cellular process | | 17 | 0.000529 | BST2,CCL8,CXCL10,GBP1,IFI16,IFI27,IFI6,IFIT1,IRF7,ISG15,MX1,RSAD2,SERPING1,SOCS1,SP100,STAT1,XAF1 | |  |
| GO:0050896 | response to stimulus | | 22 | 0.000605 | BST2,CCL8,CEACAM1,GBP1,HERC5,IFI16,IFI27,IFI44,IFI44L,IFI6,IFIH1,IFIT1,ISG15,MX1,RSAD2,SERPING1,SP100,STAT1,TRIM38,UBE2L6,UNC93B1,XAF1 | |  |
| GO:0065009 | regulation of molecular function | | 14 | 0.000889 | BST2,CASP1,CCL8,CXCL10,EIF2AK2,HERC5,IFI16,IFI27,IFI6,IFIT1,SERPING1,SOCS1,SP100,TRIM38 | |  |
| GO:0016032 | viral process | | 8 | 0.00111 | EIF2AK2,IFIH1,IFIT1,IRF7,ISG15,RSAD2,SP100,STAT1 | |  |
| GO:0042221 | response to chemical | | 16 | 0.00142 | BST2,CCL8,GBP1,HERC5,IFI27,IFI6,IFIT1,IRF7,ISG15,MX1,RSAD2,SP100,STAT1,TRIM38,UBE2L6,XAF1 | |  |
| GO:0048585 | negative regulation of response to stimulus | | 10 | 0.00143 | BST2,EIF2AK2,GBP1,IFI16,IFI6,IFIT1,SERPING1,SOCS1,STAT1,TRIM38 | |  |
| GO:0048583 | regulation of response to stimulus | | 15 | 0.00177 | BST2,CASP1,CXCL10,EIF2AK2,GBP1,HERC5,IFI16,IFI6,IFIH1,IFIT1,RSAD2,SERPING1,SP100,STAT1,UNC93B1 | |  |
| GO:0001819 | positive regulation of cytokine production | | 6 | 0.00198 | CASP1,EIF2AK2,IFI16,IFIH1,IRF7,RSAD2 | |  |
| GO:0009892 | negative regulation of metabolic process | | 13 | 0.00207 | BST2,EIF2AK2,GBP1,IFI16,IFI27,IFI6,IFIT1,IRF7,ISG15,SERPING1,SOCS1,SP100,STAT1 | |  |
| GO:0045088 | regulation of innate immune response | | 6 | 0.00207 | IFI16,IFIH1,SERPING1,SOCS1,STAT1,UNC93B1 | |  |
| GO:0050776 | regulation of immune response | | 8 | 0.00211 | BST2,IFI16,IFIH1,RSAD2,SERPING1,SOCS1,STAT1,UNC93B1 | |  |
| GO:0002684 | positive regulation of immune system process | | 8 | 0.00238 | CCL8,CXCL10,IFI16,IFIH1,ISG15,RSAD2,SERPING1,UNC93B1 | |  |
| GO:0052548 | regulation of endopeptidase activity | | 6 | 0.00254 | BST2,CASP1,IFI16,IFI27,IFI6,SERPING1 | |  |
| GO:0050790 | regulation of catalytic activity | | 12 | 0.00321 | BST2,CASP1,CCL8,CXCL10,EIF2AK2,HERC5,IFI16,IFI27,IFI6,IFIT1,SERPING1,SOCS1 | |  |
| GO:0010605 | negative regulation of macromolecule metabolic process | | 12 | 0.0034 | BST2,EIF2AK2,GBP1,IFI16,IFI27,IFI6,IRF7,ISG15,SERPING1,SOCS1,SP100,STAT1 | |  |
| GO:0031324 | negative regulation of cellular metabolic process | | 12 | 0.00362 | BST2,EIF2AK2,GBP1,IFI16,IFI27,IFI6,IRF7,ISG15,SERPING1,SOCS1,SP100,STAT1 | |  |
| GO:0034242 | negative regulation of syncytium formation by plasma membrane fusion | | 2 | 0.00382 | CXCL10,STAT1 | |  |
| GO:0060338 | regulation of type I interferon-mediated signaling pathway | | 3 | 0.0051 | IRF7,SOCS1,STAT1 | |  |
| GO:0032648 | regulation of interferon-beta production | | 3 | 0.00543 | IFIH1,IRF7,TRIM38 | |  |
| GO:0039528 | cytoplasmic pattern recognition receptor signaling pathway in response to virus | | 2 | 0.00552 | IFIH1,IRF7 | |  |
| GO:0032269 | negative regulation of cellular protein metabolic process | | 8 | 0.00649 | BST2,EIF2AK2,GBP1,IFI16,IFI6,ISG15,SERPING1,SOCS1 | |  |
| GO:0046719 | regulation by virus of viral protein levels in host cell | | 2 | 0.0127 | IFIT1,STAT1 | |  |
| GO:0032268 | regulation of cellular protein metabolic process | | 11 | 0.0206 | BST2,CASP1,CXCL10,EIF2AK2,GBP1,HERC5,IFI16,IFI27,IFI6,ISG15,SERPING1 | |  |
| GO:0030522 | intracellular receptor signaling pathway | | 4 | 0.023 | CASP1,IFIH1,IRF7,SP100 | |  |
| GO:0048584 | positive regulation of response to stimulus | | 10 | 0.024 | BST2,CASP1,CXCL10,EIF2AK2,IFI16,IFIH1,RSAD2,SERPING1,TRIM38,UNC93B1 | |  |
| GO:0050688 | regulation of defense response to virus | | 4 | 0.0245 | EIF2AK2,HERC5,IFIT1,TRIM38 | |  |
| GO:0032101 | regulation of response to external stimulus | | 7 | 0.025 | CASP1,CXCL10,EIF2AK2,HERC5,IFIT1,SERPING1,TRIM38 | |  |
| GO:0060341 | regulation of cellular localization | | 8 | 0.025 | BST2,CASP1,CXCL10,GBP1,IFI27,RSAD2,SOCS1,SP100 | |  |
| GO:0032727 | positive regulation of interferon-alpha production | | 2 | 0.0338 | IFIH1,IRF7 | |  |
| GO:0032481 | positive regulation of type I interferon production | | 3 | 0.0344 | IFI16,IFIH1,IRF7 | |  |
| GO:0051346 | negative regulation of hydrolase activity | | 5 | 0.0345 | BST2,IFI16,IFI6,IFIT1,SERPING1 | |  |
| GO:0051336 | regulation of hydrolase activity | | 8 | 0.0346 | BST2,CASP1,EIF2AK2,IFI16,IFI27,IFI6,IFIT1,SERPING1 | |  |
| GO:0032446 | protein modification by small protein conjugation | | 6 | 0.0396 | HERC5,IFIH1,SOCS1,SP100,TRIM38,UBE2L6 | |  |
| GO:2000116 | regulation of cysteine-type endopeptidase activity | | 4 | 0.0404 | CASP1,IFI16,IFI27,IFI6 | |  |
| GO:0002253 | activation of immune response | | 5 | 0.0427 | IFI16,IFIH1,IRF7,SERPING1,UNC93B1 | |  |
| GO:0043122 | regulation of I-kappaB kinase/NF-kappaB signaling | | 4 | 0.0447 | BST2,CASP1,STAT1,TRIM38 | |  |
| GO:0010951 | negative regulation of endopeptidase activity | | 4 | 0.0487 | BST2,IFI16,IFI6,SERPING1 | |  |
| GO:0035455 | response to interferon-alpha | | 2 | 0.0487 | BST2,EIF2AK2 | |  |
| **KEGG Pathways** | | | | | |  |  |
| pathway ID | | pathway description | count in gene set | false discovery rate | **Elements** |  |  |
| 5164 | | Influenza A | 8 | 3.06E-08 | CASP1,CXCL10,EIF2AK2,IFIH1,IRF7,MX1,RSAD2,STAT1 |  |  |
| 5168 | | Herpes simplex infection | 7 | 9.07E-07 | EIF2AK2,IFIH1,IFIT1,IRF7,SP100,STAT1,TAP1 |  |  |
| 5162 | | Measles | 5 | 0.00013 | EIF2AK2,IFIH1,IRF7,MX1,STAT1 |  |  |
| 4622 | | RIG-I-like receptor signaling pathway | 4 | 0.00025 | CXCL10,IFIH1,IRF7,ISG15 |  |  |
| 5160 | | Hepatitis C | 4 | 0.00232 | EIF2AK2,IFIT1,IRF7,STAT1 |  |  |
| 4623 | | Cytosolic DNA-sensing pathway | 3 | 0.00511 | CASP1,CXCL10,IRF7 |  |  |
| 4620 | | Toll-like receptor signaling pathway | 3 | 0.0195 | CXCL10,IRF7,STAT1 |  |  |
| 5161 | | Hepatitis B | 3 | 0.0431 | IFIH1,IRF7,STAT1 |  |  |

**Table S8. IFN-Score A elements biological functions obtained from GO; Geneontology and KEGG; Kyoto Encyclopedia of Genes and Genomes Databases.**

| **Biological Process (GO)** | | | | |  |
| --- | --- | --- | --- | --- | --- |
| **Pathway ID** | **pathway description** | **count in gene set** | **false discovery rate** | **Elements** |  |
| GO:0009615 | response to virus | 9 | 4.82E-12 | CCL8, CXCL10, GBP1, IFI44, IFI44L, IFIT1, IRF7, ISG15, RSAD2 |  |
| GO:0051607 | defense response to virus | 7 | 2.46E-09 | CXCL10, GBP1, IFI44L, IFIT1, IRF7, ISG15, RSAD2 |  |
| GO:0060337 | type I interferon signaling pathway | 6 | 2.46E-09 | IFI27, IFIT1, IRF7, ISG15, RSAD2, XAF1 |  |
| GO:0071357 | cellular response to type I interferon | 6 | 2.46E-09 | IFI27, IFIT1, IRF7, ISG15, RSAD2, XAF1 |  |
| GO:0019221 | cytokine-mediated signaling pathway | 8 | 9.32E-09 | CXCL10, GBP1, IFI27, IFIT1, IRF7, ISG15, RSAD2, XAF1 |  |
| GO:0071345 | cellular response to cytokine stimulus | 8 | 9.64E-08 | CXCL10, GBP1, IFI27, IFIT1, IRF7, ISG15, RSAD2, XAF1 |  |
| GO:0051707 | response to other organism | 8 | 4.15E-07 | CCL8, GBP1, IFI44,IFI44L,IFIT1,IRF7,ISG15,RSAD2 |  |
| GO:0006955 | immune response | 9 | 1.62E-06 | CCL8, CXCL10, GBP1,IFI27,IFI44L,IFIT1,ISG15,RSAD2,XAF1 |  |
| GO:0006952 | defense response | 9 | 2.72E-06 | CCL8, GBP1,IFI27,IFI44L,IFIT1,IRF7,ISG15,RSAD2,XAF1 |  |
| GO:0002252 | immune effector process | 6 | 4.46E-05 | CXCL10, GBP1, IFI44L,IFIT1,ISG15,RSAD2 |  |
| GO:0002376 | immune system process | 9 | 4.69E-05 | CCL8, CEACAM1, GBP1, IFI27,IFI44L,IFIT1,ISG15,RSAD2,XAF1 |  |
| GO:0007166 | cell surface receptor signaling pathway | 9 | 6.05E-05 | CEACAM1, CXCL10,GBP1,IFI27,IFIT1,IRF7,ISG15,RSAD2,XAF1 |  |
| GO:0045087 | innate immune response | 7 | 0.000118 | GBP1,IFI27,IFIT1,IRF7,ISG15,RSAD2,XAF1 |  |
| GO:0070887 | cellular response to chemical stimulus | 9 | 0.000134 | CCL8,CXCL10,GBP1,IFI27,IFIT1,IRF7,ISG15,RSAD2,XAF1 |  |
| GO:0006950 | response to stress | 10 | 0.000203 | CCL8,CEACAM1,GBP1,IFI27,IFI44L,IFIT1,IRF7,ISG15,RSAD2,XAF1 |  |
| GO:0045071 | negative regulation of viral genome replication | 3 | 0.00121 | IFIT1,ISG15,RSAD2 |  |
| GO:0007165 | signal transduction | 10 | 0.00279 | CCL8,CEACAM1,CXCL10,GBP1,IFI27,IFIT1,IRF7,ISG15,RSAD2,XAF1 |  |
| GO:0044700 | single organism signaling | 10 | 0.00457 | CCL8,CEACAM1,CXCL10,GBP1,IFI27,IFIT1,IRF7,ISG15,RSAD2,XAF1 |  |
| GO:0007154 | cell communication | 10 | 0.00525 | CCL8,CEACAM1,CXCL10,GBP1,IFI27,IFIT1,IRF7,ISG15,RSAD2,XAF1 |  |
| GO:0048523 | negative regulation of cellular process | 9 | 0.00759 | CCL8,CXCL10,GBP1,IFI27,IFIT1,IRF7,ISG15,RSAD2,XAF1 |  |
| GO:0002682 | regulation of immune system process | 6 | 0.0134 | CCL8,CXCL10,GBP1,IFIT1,ISG15,RSAD2 |  |
| GO:0010033 | response to organic substance | 7 | 0.0336 | GBP1,IFI27,IFIT1,IRF7,ISG15,RSAD2,XAF1 |  |
| GO:0001817 | regulation of cytokine production | 4 | 0.0473 | GBP1,IRF7,ISG15,RSAD2 |  |
| GO:0042221 | response to chemical | 8 | 0.0473 | CCL8,GBP1,IFI27,IFIT1,IRF7,ISG15,RSAD2,XAF1 |  |
| **KEGG Pathways** | | | | | |
| pathway ID | pathway description | count in gene set | false discovery rate | **Elements** | |
| 4622 | RIG-I-like receptor signaling pathway | 3 | 0.00227 | CXCL10,IRF7,ISG15 | |
| 5164 | Influenza A | 3 | 0.0157 | CXCL10,IRF7,RSAD2 | |

**Table S9. IFN-Score B elements biological functions obtained from GO; Geneontology and KEGG; Kyoto Encyclopedia of Genes and Genomes Databases.**

| **Biological Process (GO)** | | | | |  |
| --- | --- | --- | --- | --- | --- |
| Pathway ID | pathway description | count in gene set | false discovery rate | **Elements** |  |
| GO:0045087 | innate immune response | 10 | 2.03E-07 | BST2,IFI16,IFIH1,SERPING1,SOCS1,SP100,STAT1,TRIM38,UBE2L6,UNC93B1 |  |
| GO:0034341 | response to interferon-gamma | 5 | 2.15E-05 | BST2,SOCS1,SP100,STAT1,TRIM38 |  |
| GO:0002376 | immune system process | 10 | 5.51E-05 | BST2,IFI16,IFIH1,LAMP3,SERPING1,SOCS1,SP100,STAT1,TRIM38,UBE2L6 |  |
| GO:0043901 | negative regulation of multi-organism process | 5 | 5.90E-05 | BST2,IFI16,SP100,STAT1,TRIM38 |  |
| GO:0051607 | defense response to virus | 5 | 7.66E-05 | BST2,IFI16,IFIH1,STAT1,UNC93B1 |  |
| GO:0045088 | regulation of innate immune response | 6 | 9.66E-05 | IFI16,IFIH1,SERPING1,SOCS1,STAT1,UNC93B1 |  |
| GO:0019221 | cytokine-mediated signaling pathway | 6 | 0.000109 | BST2,SOCS1,SP100,STAT1,TRIM38,UBE2L6 |  |
| GO:0060333 | interferon-gamma-mediated signaling pathway | 4 | 0.000109 | SOCS1,SP100,STAT1,TRIM38 |  |
| GO:0060337 | type I interferon signaling pathway | 4 | 0.000109 | BST2,SOCS1,SP100,STAT1 |  |
| GO:0071357 | cellular response to type I interferon | 4 | 0.000109 | BST2,SOCS1,SP100,STAT1 |  |
| GO:0031347 | regulation of defense response | 7 | 0.000124 | IFI16,IFIH1,SERPING1,SOCS1,STAT1,TRIM38,UNC93B1 |  |
| GO:0002252 | immune effector process | 6 | 0.00014 | BST2,IFI16,IFIH1,SERPING1,STAT1,UNC93B1 |  |
| GO:0048525 | negative regulation of viral process | 4 | 0.000173 | BST2,IFI16,SP100,STAT1 |  |
| GO:0050776 | regulation of immune response | 7 | 0.000184 | BST2,IFI16,IFIH1,SERPING1,SOCS1,STAT1,UNC93B1 |  |
| GO:0071346 | cellular response to interferon-gamma | 4 | 0.000225 | SOCS1,SP100,STAT1,TRIM38 |  |
| GO:0002682 | regulation of immune system process | 8 | 0.000264 | BST2,IFI16,IFIH1,SERPING1,SOCS1,STAT1,TRIM38,UNC93B1 |  |
| GO:0001817 | regulation of cytokine production | 6 | 0.000292 | BST2,IFI16,IFIH1,SOCS1,TRIM38,UBE2L6 |  |
| GO:0071345 | cellular response to cytokine stimulus | 6 | 0.000304 | BST2,SOCS1,SP100,STAT1,TRIM38,UBE2L6 |  |
| GO:0032479 | regulation of type I interferon production | 4 | 0.000526 | IFI16,IFIH1,TRIM38,UBE2L6 |  |
| GO:0051239 | regulation of multicellular organismal process | 9 | 0.000967 | BST2,IFI16,IFIH1,SERPING1,SOCS1,SP100,STAT1,TRIM38,UBE2L6 |  |
| GO:0051707 | response to other organism | 6 | 0.000984 | BST2,IFI16,IFIH1,SOCS1,STAT1,UNC93B1 |  |
| GO:0045069 | regulation of viral genome replication | 3 | 0.00483 | BST2,IFI16,TRIM38 |  |
| GO:0051241 | negative regulation of multicellular organismal process | 6 | 0.00603 | BST2,IFIH1,SERPING1,SP100,STAT1,UBE2L6 |  |
| GO:1903901 | negative regulation of viral life cycle | 3 | 0.00667 | BST2,IFI16,SP100 |  |
| GO:0002698 | negative regulation of immune effector process | 3 | 0.0101 | BST2,SERPING1,TRIM38 |  |
| GO:0032446 | protein modification by small protein conjugation | 5 | 0.0142 | IFIH1,SOCS1,SP100,TRIM38,UBE2L6 |  |
| GO:0050777 | negative regulation of immune response | 3 | 0.0177 | BST2,IFI16,SERPING1 |  |
| GO:0035456 | response to interferon-beta | 2 | 0.0203 | BST2,STAT1 |  |
| GO:0002683 | negative regulation of immune system process | 4 | 0.0224 | BST2,IFI16,SERPING1,TRIM38 |  |
| GO:0031348 | negative regulation of defense response | 3 | 0.026 | IFI16,SERPING1,TRIM38 |  |
| GO:0048585 | negative regulation of response to stimulus | 6 | 0.026 | BST2,IFI16,SERPING1,SOCS1,STAT1,TRIM38 |  |
| GO:0060397 | JAK-STAT cascade involved in growth hormone signaling pathway | 2 | 0.0334 | SOCS1,STAT1 |  |
| GO:0060334 | regulation of interferon-gamma-mediated signaling pathway | 2 | 0.0357 | SOCS1,STAT1 |  |
| GO:0002253 | activation of immune response | 4 | 0.0369 | IFI16,IFIH1,SERPING1,UNC93B1 |  |
| GO:0060396 | growth hormone receptor signaling pathway | 2 | 0.0394 | SOCS1,STAT1 |  |
| GO:0071378 | cellular response to growth hormone stimulus | 2 | 0.0417 | SOCS1,STAT1 |  |
| GO:0051704 | multi-organism process | 7 | 0.0433 | BST2,IFI16,IFIH1,SOCS1,SP100,STAT1,UNC93B1 |  |
| **KEGG Pathways** | | | | | |
| pathway ID | pathway description | count in gene set | false discovery rate | **Elements** | |
| 5168 | Herpes simplex infection | 4 | 0.00136 | IFIH1,SP100,STAT1,TAP1 | |

**Supplement References**

1 Kaiser, H. F. An index of factorial simplicity. *Psychometrika* **39**, 31-36, doi:10.1007/bf02291575 (1974).

2 Szklarczyk, D. *et al.* STRING v10: protein-protein interaction networks, integrated over the tree of life. *Nucleic Acids Res* **43**, D447-452, doi:10.1093/nar/gku1003 (2015).
